# Vascular endothelial growth factor from retinal pigment epithelium is essential in choriocapillaris and axial length maintenance

**DOI:** 10.1093/pnasnexus/pgac166

**Published:** 2022-08-24

**Authors:** Yan Zhang, Heonuk Jeong, Kiwako Mori, Shin-Ichi Ikeda, Chiho Shoda, Yukihiro Miwa, Ayaka Nakai, Junhan Chen, Ziyan Ma, Xiaoyan Jiang, Hidemasa Torii, Yoshiaki Kubota, Kazuno Negishi, Toshihide Kurihara, Kazuo Tsubota

**Affiliations:** Laboratory of Photobiology, Keio University School of Medicine, 35 Shinanomachi, Shinjuku-ku, Tokyo 160-8582, Japan; Department of Ophthalmology, Keio University School of Medicine, 35 Shinanomachi, Shinjuku-ku, Tokyo 160-8582, Japan; Laboratory of Photobiology, Keio University School of Medicine, 35 Shinanomachi, Shinjuku-ku, Tokyo 160-8582, Japan; Department of Ophthalmology, Keio University School of Medicine, 35 Shinanomachi, Shinjuku-ku, Tokyo 160-8582, Japan; Laboratory of Photobiology, Keio University School of Medicine, 35 Shinanomachi, Shinjuku-ku, Tokyo 160-8582, Japan; Department of Ophthalmology, Keio University School of Medicine, 35 Shinanomachi, Shinjuku-ku, Tokyo 160-8582, Japan; Laboratory of Photobiology, Keio University School of Medicine, 35 Shinanomachi, Shinjuku-ku, Tokyo 160-8582, Japan; Department of Ophthalmology, Keio University School of Medicine, 35 Shinanomachi, Shinjuku-ku, Tokyo 160-8582, Japan; Laboratory of Photobiology, Keio University School of Medicine, 35 Shinanomachi, Shinjuku-ku, Tokyo 160-8582, Japan; Department of Ophthalmology, Keio University School of Medicine, 35 Shinanomachi, Shinjuku-ku, Tokyo 160-8582, Japan; Department of Ophthalmology, Nihon University School of Medicine, 30-1 Oyaguchikamicho, Itabashi City, Tokyo 173-8610, Japan; Laboratory of Photobiology, Keio University School of Medicine, 35 Shinanomachi, Shinjuku-ku, Tokyo 160-8582, Japan; Department of Ophthalmology, Keio University School of Medicine, 35 Shinanomachi, Shinjuku-ku, Tokyo 160-8582, Japan; Aichi Animal Eye Clinic, 3 Chome-17-3 Honjitori, Minami Ward, Nagoya, Aichi 457-0074, Japan; Laboratory of Photobiology, Keio University School of Medicine, 35 Shinanomachi, Shinjuku-ku, Tokyo 160-8582, Japan; Department of Ophthalmology, Keio University School of Medicine, 35 Shinanomachi, Shinjuku-ku, Tokyo 160-8582, Japan; Department of Ophthalmology, Nihon University School of Medicine, 30-1 Oyaguchikamicho, Itabashi City, Tokyo 173-8610, Japan; Laboratory of Photobiology, Keio University School of Medicine, 35 Shinanomachi, Shinjuku-ku, Tokyo 160-8582, Japan; Department of Ophthalmology, Keio University School of Medicine, 35 Shinanomachi, Shinjuku-ku, Tokyo 160-8582, Japan; Laboratory of Photobiology, Keio University School of Medicine, 35 Shinanomachi, Shinjuku-ku, Tokyo 160-8582, Japan; Department of Ophthalmology, Keio University School of Medicine, 35 Shinanomachi, Shinjuku-ku, Tokyo 160-8582, Japan; Laboratory of Photobiology, Keio University School of Medicine, 35 Shinanomachi, Shinjuku-ku, Tokyo 160-8582, Japan; Department of Ophthalmology, Keio University School of Medicine, 35 Shinanomachi, Shinjuku-ku, Tokyo 160-8582, Japan; Laboratory of Photobiology, Keio University School of Medicine, 35 Shinanomachi, Shinjuku-ku, Tokyo 160-8582, Japan; Department of Ophthalmology, Keio University School of Medicine, 35 Shinanomachi, Shinjuku-ku, Tokyo 160-8582, Japan; Department of Anatomy, Keio University School of Medicine, 35 Shinanomachi, Shinjuku-ku, Tokyo 160-8582, Japan; Department of Ophthalmology, Keio University School of Medicine, 35 Shinanomachi, Shinjuku-ku, Tokyo 160-8582, Japan; Laboratory of Photobiology, Keio University School of Medicine, 35 Shinanomachi, Shinjuku-ku, Tokyo 160-8582, Japan; Department of Ophthalmology, Keio University School of Medicine, 35 Shinanomachi, Shinjuku-ku, Tokyo 160-8582, Japan; Department of Ophthalmology, Keio University School of Medicine, 35 Shinanomachi, Shinjuku-ku, Tokyo 160-8582, Japan; Tsubota Laboratory Inc., 34 Shinanomachi, 304 Toshin Shinanomachi Ekimae Building, Shinjuku-ku, Tokyo 160-0016, Japan

**Keywords:** myopia, VEGF, choriocapillaris, axial length

## Abstract

Myopia, which prevalence is rapidly increasing, causes visual impairment; however, the onset mechanism of pathological axial length (AL) elongation remains unclear. A highly vascularized choroid between the retinal pigment epithelium (RPE) and sclera not only maintains physiological activities, but also contributes to ocular development and growth regulation. Vascular endothelial growth factor (VEGF) secreted from the RPE to the choroid is essential for retinal function and maintenance of the choriocapillaris. Herein, we demonstrated that the loss of VEGF secreted from the RPE caused abnormal choriocapillaris development and AL elongation, with features similar to those of the lens-induced myopia (LIM) mouse model, whereas VEGF overexpression by knocking-out von Hippel–Lindau (VHL) specific to the RPE expands the choriocapillaris and shortens the AL. Additionally, LDL Receptor Related Protein 2 (LRP2) deletion in the RPE downregulated VEGF expression and leads to pathological AL elongation. Furthermore, high-myopia patients without choriocapillaris demonstrated longer ALs than did those with preserved choriocapillaris. These results suggest that physiological secretion of VEGF from the RPE is required for proper AL development by maintaining the choriocapillaris. The pinpoint application of VEGF to the choriocapillaris may become a potential intervention for the prevention and treatment of axial myopia progression.

Significance Statement Mechanism of myopia onset remains unclear. Here we show that loss of VEGF secreted from RPE causes abnormal choriocapillaris development and axial length elongation, appearing the features like the LIM mouse model, whereas VEGF overexpression by knocking out VHL specific in RPE expands choriocapillaris and shorten AL. Also, LRP2 deletion in RPE downregulates VEGF expression and shows pathological AL elongation. Furthermore, high myopic patients without choriocapillaris showed longer AL than those who have preserved choriocapillaris. These results suggest that physiological secretion of VEGF from RPE is required for the proper development of AL by maintaining choriocapillaris. The pinpoint application of VEGF to the choriocapillaris may become a potential intervention for the prevention and treatment of axial myopia progression.

## Introduction

Myopia, one of the most common eye disorders, is a condition in which light is focused in front of the retina due to eyeball elongation. Generally, the visual acuity of patients with myopia can be easily corrected; however, structural changes in the eyeballs in high-myopia increase the risk of pathologic ocular complications such as cataracts, glaucoma, retinal detachment, and myopic macular degeneration, all of which can result in irreversible vision loss (1). The prevalence of myopia has risen dramatically in recent decades and is anticipated to affect 50% of the global population by 2050 (2). Furthermore, the COVID-19 pandemic outbreak has resulted in increased digital device time and near work; however, limited outdoor activities due to the lockdown and quarantine (3). According to some scientists, this lifestyle change will exacerbate the myopic boom. However, the mechanism of the onset of myopia remains unknown. One hypothesis is that blurred retinal vision triggers a retinoscleral signaling cascade, which influences the retinal pigment epithelial (RPE) physiology and choroidal thickness (4–6), contributing to ocular refractive change in the short term, and then alters the extracellular matrix remodeling in the sclera (7), thus contributing to eye size change in the long term.

The choroid is a highly vascular structure located between the RPE and the sclera that plays multiple important roles in the maintenance of ocular physiological activities (8, 9). It also provides nutrients to the retina, regulates the ocular temperature, controls the intraocular pressure (IOP), and absorbs light that could harm vision. Regarding ocular development, the choroid contributes to eye growth regulation (10) and, more importantly, choroidal accommodation adjusts the increase or decrease of the thickness of the choroid to allow the eye to focus on an image when the retina detects a sign of defocus (5, 11). Existing animal models, such as mice (12), chicks (5, 11, 13), marmosets (14), rhesus monkeys (15), and guinea pigs (16), have demonstrated changes in choroidal thickness in response to imposed retinal defocus. Whereas, hyperopic defocus reduced choroidal thickness and increased ocular elongation, myopic defocus increased choroidal thickness and inhibited ocular elongation. Furthermore, clinical studies have shown that choroidal thickness decreases significantly in high-myopia patients (17, 18), and that subfoveal choroidal thickness is negatively correlated with axial length (AL) (19). Consequently, an increasing number of scientists believe that choroidal thickness can be used as a clinical biomarker for the onset and progression of myopia (20, 21).

Aside from choroidal thinning in high myopic patients, aqueous levels of VEGF in patients with high-myopia were significantly lower than in controls (22). RPE, which is a major source of VEGF (6), is also one of the key tissues involved in the retinoscleral signaling cascade. Previous studies have also demonstrated that VEGF secreted by RPE cells is important for the development and maintenance of the choriocapillaris (23–26). Identifying how the RPE and/or choroid influence eye growth may lead to new myopia control methods.

The *Lrp2* conditional gene KO mice, a high myopic animal model that has been used in many myopic studies (27–29), was used in this study to reveal that RPE cells are the key factors for proper ocular development. Based on abnormal choriocapillaris development and decreased *Vegf* expression in RPE cells in *Lrp2* conditional gene KO mice, we used *von Hippel–Lindau* (*Vhl*) and *Vegf* RPE-specific KO mice compared to lens-induced myopia (LIM) mice (30) to confirm that VEGF plays an essential role in proper choriocapillaris development and AL maintenance.

## Results

### *Lrp2* gene in RPE cells but not in retinal neurons are required for the proper ocular biometric development

*LRP2* is a protein-coding gene that encodes a transmembrane receptor binding with multiligands in various organs, including the eyes. Mutations in the *LRP2* gene lead to Donnai-Barrow/facio-oculo-acoustico-renal syndrome with a typical craniofacial feature (31). High-myopia, retinal detachment, retinal dystrophy, and progressive vision loss are ocular complications of this rare autosomal recessive inherited disease (32). Nonsense mutations in zebrafish *Lrp2* resulted in enlarged eyes and elevated IOP (33). General and specific *Lrp2* mutations in mice also resulted in enlarged eye sizes (27, 28, 29, 34), but no increase in the IOP.

**Fig. 1.**
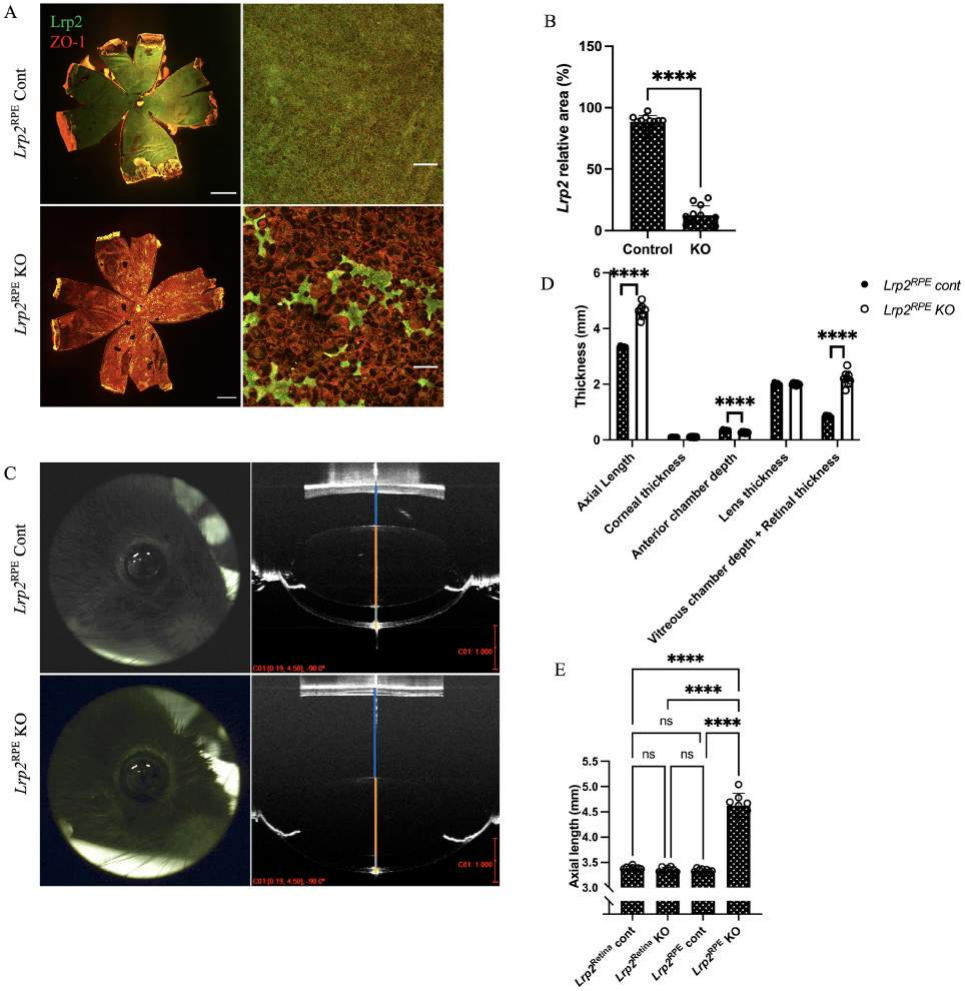
*Lrp2* gene in RPE cells but not in retinal neurons are required for the proper ocular biometric development. (A) Representative immunohistochemistry of choroid flat-mount of 8-week-old *Lrp2*^RPE^ Cont and *Lrp2*^RPE^ KO mouse (green: LRP2, red: ZO and 1). Enlargement and deformation of RPEs and less LRP2 expression are observed in *Lrp2*^RPE^ KO mouse (bottom) compared to Cont mouse (upper). Scale bar: 1 mm (left panels), 100 µm (right panels). (B) Quantification of LRP2 relative area in RPEs of the choroid of 8-week-old *Lrp2*^RPE^ KO and *Lrp2*^RPE^ Cont mouse. *n* = 3. ^****^P < 0.0001, two-tailed Student's *t*-tests. (C) Representative OCT image of the whole eye in 8-week-old *Lrp2*^RPE^ Cont and *Lrp2*^RPE^ KO mouse (vitreous chamber depth + retinal thickness; blue line, lens thickness; orange line, anterior chamber depth; gray line; corneal thickness; and yellow line). Scale bar in red: 1 mm. (D) Quantification of different axial parameters in 8-week-old *Lrp2*^RPE^ Cont and *Lrp2*^RPE^ KO mouse. Significant differences are shown in axial length, vitreous chamber depth + retinal thickness, and anterior chamber depth. *n* = 4 Cont, *n* = 8 KO. ^****^P < 0.0001, two-tailed Student's *t-*tests. (E) Axial length comparison between different cre-transgenic *Lrp2*^floxed/floxed^ mice (*Lrp2*^Retina^ Cont, *Lrp2*^Retina^ KO; *Lrp2*^RPE^ Cont, *Lrp2*^RPE^ KO). No significant difference is seen in *Lrp2*^Retina^ KO and its control (*Lrp2*^Retina^ Cont) group. *n* = 4. ^****^P < 0.0001, one-way ANOVA tests. Graphs present as mean ± SD.

To clarify the link between the *Lrp2* gene and high-myopia, a mouse line, which contains homozygous floxed *Lrp2* allele, is crossed with eighter the *Best1*-Cre or the *Chx10*-Cre transgenic mice to inactivated *Lrp2* in RPE cells (*Lrp2*^RPE^ KO) or the neural retina (*Lrp2*^Retina^ KO), respectively. Deletion of LRP2 expression in RPE cells (Fig. 1A) was confirmed by whole-mount immunohistochemical (IHC) staining with LRP2 antibody and ZO-1 antibody, which is a tight junction marker of RPE cells. In addition, conditional deletion of LRP2 in RPE cells and neural retina were confirmed by mouse eye cross sections immunohistochemical staining (Supplementary Material, Figs. S1 and S2C). LRP2 expression was significantly decreased in RPE cells of *Lrp2*^RPE^ KO mice (Fig. 1A and B, Supplementary Material, Table S1), and the RPE cells were remarkably enlarged and deformed in mutant mice (Fig. 1A). *Lrp2*^RPE^ KO mice showed enlarged and protruding eyes compared with *Lrp2*^RPE^ control, *Lrp2*^Retina^ KO and *Lrp2*^Retina^ control mice (Fig. 1C and E, Supplementary Material, Table S3). ALs of both *Lrp2*^RPE^ KO and control mice were examined by optical coherence tomography (OCT) from 3 to 40 weeks of age (Supplementary Material, Fig. S3). However, no significant differences were observed in 8-week-old *Lrp2*^Retina^ KO and control mice (Fig. 1E, Supplementary Material, Fig. S2A). *Lrp2*^RPE^ KO mice also had shallow anterior chamber depth and longer vitreous chamber depth, while no significant differences were observed in the corneal thickness or lens thickness in both conditional KO mouse lines (Fig. 1D, Supplementary Material, Table S2). Moreover, cross sections IHC staining with isolectin B4 antibody, Anti-Collagen1 antibody (Col1), and whole-mount IHC staining with ZO-1 antibody (Supplementary Material, Fig. S4A) were conducted in *Best1*-Cre and its control mice. Also, OCT measurements of *Lrp2*^floxed/–^  *Best1*-cre + mice (Supplementary Material, Figs. S4B and S4C, Table S18 and S19) were performed to confirm the *Best1*-Cre alone does not affect the experiment. OCT measurements of *Lrp2*^floxed/–^  *Chx10*-cre + mice (Supplementary Material, Fig. S2B, Table S17) were also performed to confirm the *Chx10*-cre alone does not alter the experimental outcomes. Collectively, these findings suggest that the *Lrp2* gene in RPE cells, but not neural cells in the retina, is required for proper ocular development.

### *Lrp2* ^RPE^ KO mice show abnormal choriocapillaris development and decreased expression of *Vegf* in RPE cells

**Fig. 2.**
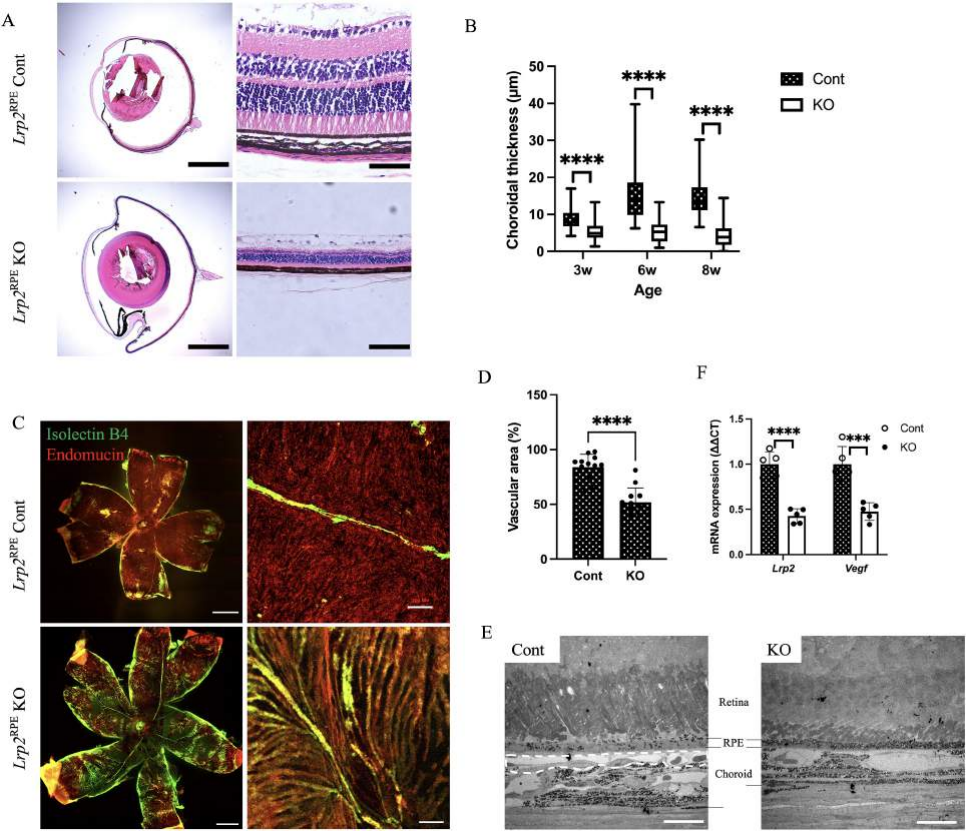
Decrease of *Vegf* expression from RPEs and abnormal development of choriocapillaris are observed in *Lrp2*^RPE^ KO mice. (A) The chordal region in the representative H&E-stained cross-sections of enucleated eyes from 6-week-old *Lrp2*^RPE^ Cont and *Lrp2*^RPE^ KO mouse showing enlarged overall eye size and thinner choroidal thickness in KO mouse (bottom). Scale bar: 1 mm (left panels), 50 µm (right panels). (B) Choroidal thickness measurements in 3-, 6-, and 8-week-old *Lrp2*^RPE^ Cont and *Lrp2*^RPE^ KO mouse showing reduction of choroidal thickness in KO mouse. (C) Representative immunohistochemistry of choroid flat-mount (green: isolectin B4, red: endomucin) and (D) quantification of vascular area in choroid of 8-week-old *Lrp2*^RPE^ Cont and *Lrp2*^RPE^ KO mouse showing reduction of choriocapillaris in KO mouse. Scale bar: 1 mm (left panels), 20 µm (right panels). (E) Transmission electron microscope observation of choroids of 8-week-old *Lrp2*^RPE^ Cont and *Lrp2*^RPE^ KO mouse. Choriocapillaris highlighted by dashed line in Cont (left) is disappeared in KO (right), resulting in choroidal thinning. Scale bar: 100 µm. (F) *Lrp2* and *Vegf* mRNA expression from primary RPEs in 6-week-old *Lrp2*^RPE^ Cont and *Lrp2*^RPE^ KO mouse. Graphs represent as mean ± SD. *n* = 5. ***P < 0.001, ^****^P < 0.0001, two-tailed Student's *t*-tests.

Histological cross-sections of enucleated eyes from 6-week-old mice demonstrate enlarged overall eye size with thinner and denser retina, choroid, and sclera in *Lrp2*^RPE^ KO mice (Fig. 2A). Hematoxylin and eosin (H&E) staining of the paraffin sections revealed that the thickness of the whole retinal layer, outer nuclear layer (ONL), and inner nuclear layer (INL) (Supplementary Material, Fig. S5A to C, Table S20 to 22), and choroidal (Fig. 2B, Supplementary Material, Table S4) and scleral layer (Supplementary Material, Fig. S5D, Table S23) were significantly decreased in the mutant eyes from 3 to 8 weeks of age. In addition to the thinning of the retinal layer, the number of retinal vessel branches was also reduced in the KO mice (Supplementary Material, Fig. S6A and B, Table S24).

To investigate choroidal vascularization in *Lrp2*^RPE^ KO mice, we stained choroidal whole-mounts with endomucin antibody, a specific marker for choriocapillaris, and isolectin B4, a marker for medium/larger-sized blood vessels. *Lrp2*^RPE^ KO mice showed choroidal hypoplasia, while dense and curled choriocapillaris was observed in the control mice (Fig. 2C and D). In addition, choroidal vascularization in *Lrp2*^Retina^ KO mice were also confirmed by choroidal whole-mount IHC staining (Supplementary Material, Fig. S2D). To further confirm the choroidal structure in *Lrp2*^RPE^ KO mice, sections of KO and control mice were examined using transmission electron microscopy (TEM). Besides the atrophy of photoreceptor layers, which has been previously described (27), the choriocapillaris beneath Brush's membrane disappeared; instead, medium/larger-sized choroidal blood vessels and melanocytes moved up in KO mice were observed by TEM (Fig. 2E).

VEGF released from RPE cells is essential for the development and maintenance of choriocapillaris (23–25). We detected the mRNA expression of RPE cells in *Lrp2*^RPE^ KO mice. Mutant mice not only exhibited a significantly lower level of *Lrp2* expression, but also a significantly reduced *Vegf* level (Fig. 2F, Supplementary Material, Table S6).

We conducted an electroretinography (ERG) examination to investigate the visual function of KO mice due to atrophy of photoreceptors and hypoplasia of the choriocapillaris. Significant reductions in both scotopic (rod-driven) and photopic (cone-driven) responses were observed in *Lrp2*^RPE^ KO mice (Supplementary Material, Fig. S7, Table S25 to 30).

### AL elongated along with the development of choroidal thickness in C57B6/J mice

**Fig. 3.**
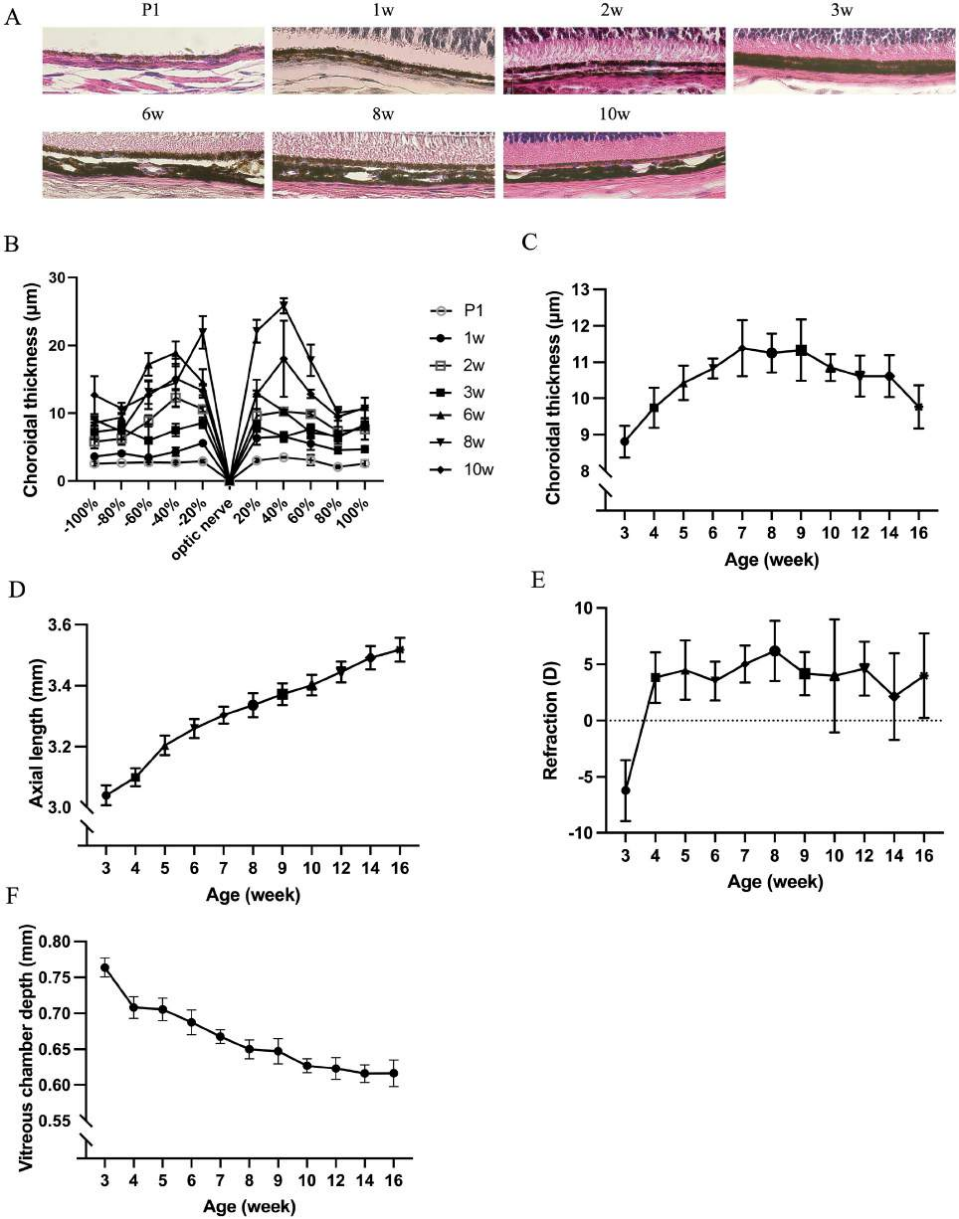
Choroidal thickness and axial length development in C57B6/J mice. (A) Representative H&E-stained cross-sections of enucleated eyes from wild-type C57B6/J mice and (B) measurement of choroidal thickness according to its distance from the optic nerve. Thickness of choroid is relatively thicker in the posterior pole than the anterior pole *n* = 3. (C) Choroidal thickness measurement by OCT images shows that choroidal thickness gradually increases and reaches a plateau at about 7 to 9 weeks old after then the thickness gradually become thinner along with the age *n* = 3. (D) Axial length, (E) refraction error, and (F) vitreous chamber depth with growth showing axial length increase with age and emmetropic refractive status. Numbers between each time point in D indicate slope *n* = 6. Graphs represent as mean ± SD.

To observe the physiological development of choroidal thickness and AL in normal wild-type mice, paraffin cross-sections of enucleated eyes from postnatal day 1 (P1) and 1-, 2-, 3-, 6-, 8-, and 10-week-old mice were stained via H&E staining (Fig. 3A). All samples were observed and quantified using a biological microscope. The choroidal thickness gradually increased from P1 and reached its peak value at approximately 8 weeks of age, and the thickness at the posterior pole was relatively thicker than the anterior pole (Fig. 3B). OCT measurements were also performed in wild-type mice from 3 to 16 weeks of age. Similar to the results of H&E-stained paraffin sections, choroidal thickness increased gradually and reached a plateau at 7 to 9 weeks of age, following which the thickness gradually decreased with age (Fig. 3C). AL elongated along with the development of choroidal thickness, and it is worth mentioning that the growth rate of AL slows down after the choroidal thickness reaches a plateau (Fig. 3D). The refraction status indicated emmetropia on average (Fig. 3E). The vitreous chamber depth, which is another important refractive parameter, decreased with age (Fig. 3F).

### *Vegf* ^RPE^ KO mice reveal myopic features while *Vhl*^RPE^ KO mice reveal hyperopic features

**Fig. 4.**
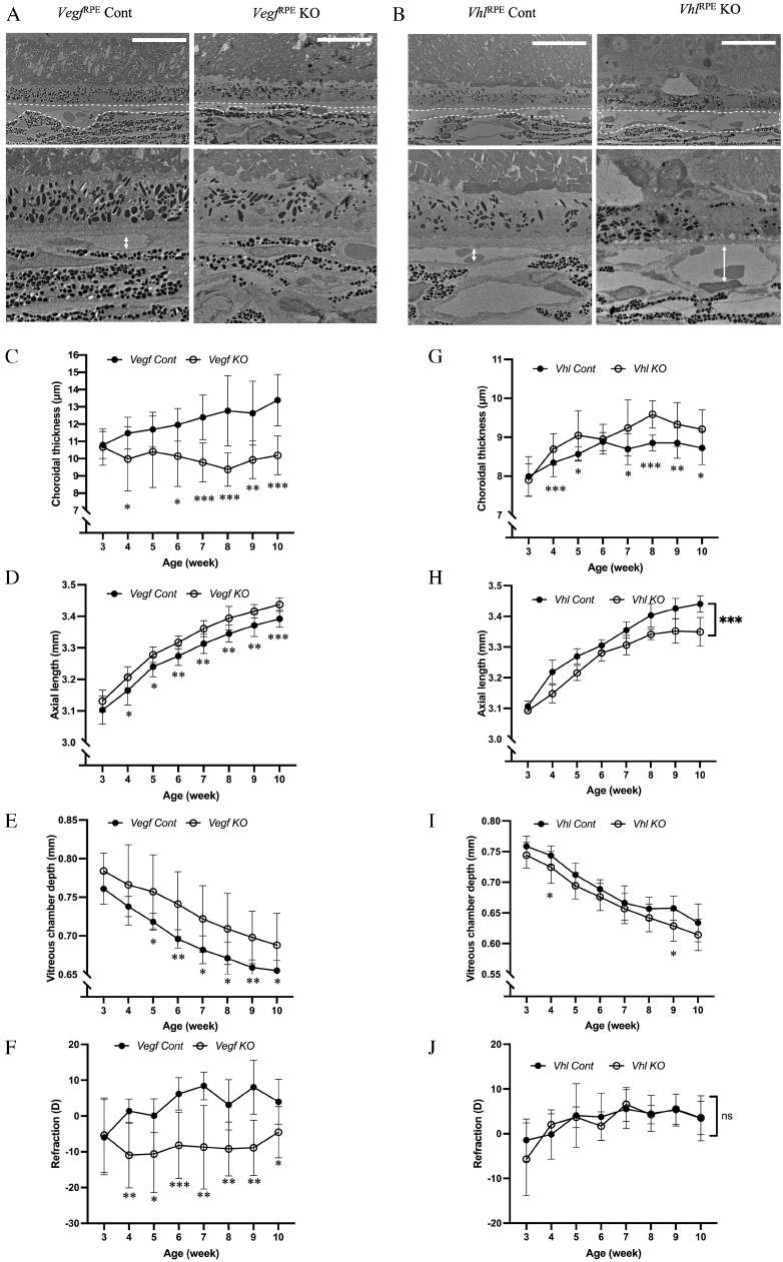
*Vegf* ^RPE^ KO mice reveal myopic features while *Vhl*^RPE^ KO mice reveal hyperopic features. (A and B) Transmission electron microscope observation of choroids in 10-week-old *Vegf*^RPE^ Cont and *Vegf*^RPE^ KO (A), and *Vhl*^RPE^ Cont and *Vhl*^RPE^ KO (B) mouse showing choriocapillaris thinning in *Vegf*^RPE^ KO and choriocapillaris vasodilatation in *Vhl*^RPE^ KO. Choriocapillaris highlighted by dashed line. Scale bar: 20 µm. (C to J) Choroidal thickness (C and G), axial length (D and H), vitreous chamber depth (E and I) and refraction error (F and J) measurement of *Vegf*^RPE^ Cont and *Vegf*^RPE^ KO (C to F), and *Vhl*^RPE^ Cont and *Vhl*^RPE^ KO mouse (G to J) with growth *n* = 5. *P < 0.05, **P < 0.01, ***P < 0.001, two-tailed Student's *t-*tests.

Other researchers have shown that knocking out *Vegf* in the RPE cells of mice leads to degeneration of the choriocapillaris (23–25), while knocking out *Vhl* in RPE cells results in increased production of VEGF and choriocapillaris vasodialation (35). To investigate the relationship between the choriocapillaris and AL, we generated *Vegf*^RPE^ KO and *Vhl*^RPE^ KO mice lines to compare their AL and refraction status. TEM was used to confirm the absence of choriocapillaris in *Vegf*^RPE^ KO mice (Fig. 4A) and choriocapillaris vasodilatation in *Vhl*^RPE^ KO mice (Fig. 4B). Thinner choroid and longer AL and vitreous chamber depth were found in *Vegf*^RPE^ KO mice compared with control mice from 3 to 10 weeks of age by OCT examinations (Fig. 4C to E, Supplementary Material, Table S7 to 9). Moreover, a myopic refraction status was observed in *Vegf*^RPE^ KO mice (Fig. 4F, Supplementary Material, Table S10). Conversely, *Vhl*^RPE^ KO mice had thicker choroid and shorter AL compared to control mice (Fig. 4G to I, Supplementary Material, Table S11 to 13). Both *Vhl*^RPE^ KO and control mice demonstrated emmetropic refraction status (Fig. 4J, Supplementary Material, Table S14), whereas *Vegf*^RPE^ KO mice showed similar myopic features to LIM mice (Supplementary Material, Fig. S8A, Table S31), which had a similar choroidal thinning and pathological AL elongation (Supplementary Material, Fig. S8B and C, Table S32 and 33). Collectively, these findings suggest that proper development of the choriocapillaris is essential for AL maintenance.

### Without choriocapillaris, AL of high-myopia increased more than those with preserved choriocapillaris

**Fig. 5.**
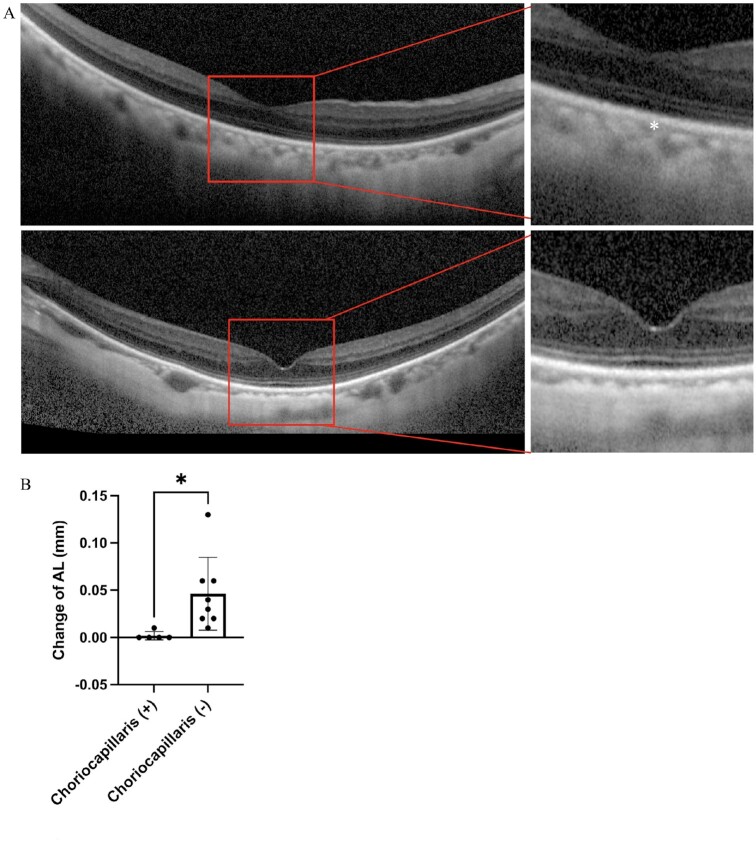
Axial length of high myopic patients without choriocapillaris increased more compared with high myopic patients who have preserved choriocapillaris. (A) OCT of a 70-year-old female high myopic patient with an axial length of 27.22 mm is shown in the upper panel of A (choriocapillaris highlighted by *), while OCT of a 70-year-old female high myopic patient with an axial length of 28.72 and absent of choriocapillaris are shown in the lower panel of A. High myopic patients with choriocapillaris degeneration [choriocapillaris (-) increased more compared with high myopic patients who have preserved choriocapillaris (+)] in a follow-up for more than 6 months. *P < 0.05, two-tailed Student's *t-*tests (B) (*n* = 5 in choriocapillaris group, *n* = 8 in choriocapillaris degeneration group, respectively). Error bars indicate mean ± SD.

It is generally assumed that the AL remains constant in adults, and an age-related decrease in the AL has been reported in several studies (36). However, we observed a continuous increase in the AL in elderly patients with high-myopia in the outpatient department of Keio University Hospital. Baseline data for these patients are shown in Supplementary Material, Table S15. To evaluate whether the choriocapillaris affects AL elongation, we compared the change in AL between high myopic patients with choriocapillaris and those with complete choriocapillaris degeneration (Supplementary Material, Table S16). In a follow-up longer than 6 months, the ALs of high-myopia patients without choriocapillaris elongated more than did those of patients with preserved choriocapillaris (Fig. 5).

## Discussion

The choroid is a multifunctional structure that plays an active role in emmetropia and myopization processes (8–10). Clinical studies have conclusively demonstrated that, in addition to age-related choroid thinning (36), subfoveal choroidal thickness decreases with increasing AL (17, 18). In our study, *Lrp2*^RPE^ KO mice, a high myopic mouse model, showed choroid denegation and a decrease in total choroidal thickness, as well as the characteristics of the previously published dramatically elongated AL and retinal degeneration (27, 34). Many animal studies, including form-deprivation myopia and LIM experiments (37), have confirmed that changes in choroidal thickness affect retinal movement (5), ensuring that the image falls on the retina accurately and thus, promotes the process of emmetropia. In our study, we measured ocular parameters that reflect the development of eye sizes, such as refractive error, AL, and choroidal thickness, in C57B6/J mice aged P10 to 10 weeks without any experimental intervention. The choroidal thickness was substantially thinned within 2 weeks after birth, the tortuous vascular structure gradually appeared with an increase in choroidal thickness, with the thickness reaching a plateau between 7 and 9 weeks, and then gradually became thinner with age. Furthermore, the choroidal thickness at the posterior pole is thicker than that at the anterior pole, which may explain the choroidal accommodation required to ensure that light is accurately focused on the retina at the posterior pole. It has also been demonstrated in clinical studies that choroidal thickness thickens and reaches a peak during adolescence (38, 39), and then gradually becomes thinner with age (40). Therefore, we hypothesize that the relatively lower choroidal thickness in the early stage ensures normal elongation of the AL and emmetropia of the eye, whereas excessive AL growth by choroidal thickening leads to myopization. Along with the normal elongation of the AL, the choroidal thickness gradually increases to prevent the AL from becoming too long and to maintain proper eye size. Furthermore, because it is widely believed that the AL remains constant in adults, this means that even without the protection of the choroid, the AL no longer grows, and the choroid gradually becomes thinner.

**Fig. 6.**
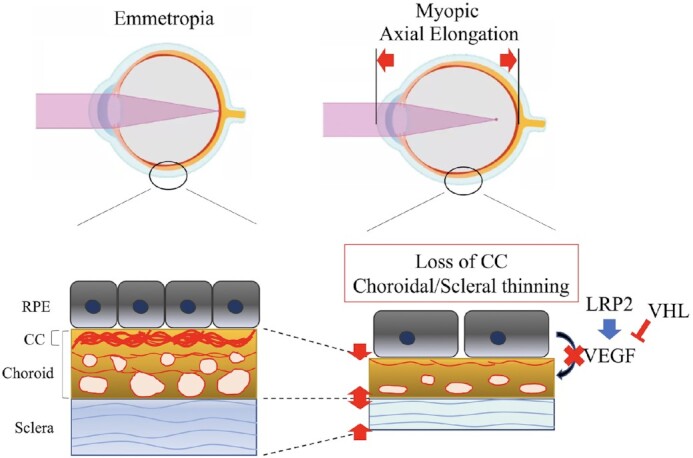
VEGF derived from RPE is necessary for choriocapillaris development and maintenance, which is essential for overall ocular size development and maintenance. The development of emmetropic eyes requires VEGF derived from RPE to promote the development of choriocapillaris and maintain their physiological thickness. The decrease of VEGF derived from RPE can cause choriocapillaris dysplasia, and the thinner choroid thickness will promote axial length elongation and result in myopia. In a word, generous physiological secretion of VEGF is essential for choriocapillaris and axial length maintenance, while in the case of hypoxia in high-myopia, local pathological secretion of VEGF results in the formation of myopic choroidal neovascularization.

Furthermore, we found that, when compared to the thickness of the entire choroid, the thickness of the choriocapillaris layer may be more closely related to AL growth. In addition to thinning the entire choroidal thickness, we observed choriocapillaris degeneration in *Lrp2*^RPE^ KO mice. Additionally, *Vegf*^RPE^ KO mice, in which choriocapillaris degeneration was also observed, demonstrated longer AL and myopic refractive status, whereas *Vhl*^RPE^ KO mice, which has a higher level of VEGF expression after gene deletion (35), showed dilatation of the choriocapillaris with shorter ALs (Fig. 6). The choroid is anatomically divided into five layers (9). However, in clinical practice, the choroid is divided into three layers: the choriocapillaris, Sattler's layer (the middle vessel layer), and Haller's layer (the large vessel layer). Previous cross-sectional studies have demonstrated that age-related choroidal thickness thinning and myopic choroidal thickness changes occur primarily in Haller's layer (40, 41). However, in the follow-up of elderly people with progressing high-myopia, while the overall choroidal thickness did not change, the AL growth was significantly higher in patients with choriocapillaris degeneration than in those without choriocapillaris degeneration. Based on the animal and clinical findings, we speculate that the presence and thickness of the choriocapillaris determine the proper physiological elongation of the AL. Without the effect of the environment, the thickness of the choroid or choriocapillaris and the size of the eyeball are controlled by the genes of each person. The AL may be longer in those with a thinner choriocapillaris than in those with a thicker choriocapillaris. Due to environmental effects, AL may expand beyond its physiological growth, and when this occurs, the large and medium vessels are compressed, and the thickness decreases.

VEGF overexpression has long been recognized as a risk factor for ocular disorders (42), including myopic choroidal neovascularization, age-related macular degeneration, and diabetic retinopathy. Anti-VEGF therapies have been applied in the treatment of these disorders for a long time. Anti-VEGF therapy led to a significant reduction in subfoveal choroidal thickness (43). Animal studies have also indicated that a single intravitreal injection of bevacizumab, an antibody raised against human VEGF, inhibits the choroidal thickening in the chicken that normally occurs during recovery from myopia (44). Therefore, a certain amount of VEGF may be required to maintain choroidal thickness. Previous clinical studies have found that the concentration of VEGF in the vitreous cavity of myopia patients is significantly lower, most likely due to the increased length of the ocular axis and enlargement of the vitreous cavity, which dilutes the concentration of VEGF (22, 45, 46). Furthermore, some researchers believe that significantly lower VEGF concentrations in myopic eyes could explain why such eyes have a lower prevalence of exudative AMD and DR (45). In our study, the concentration of *Vegf* from RPE cells decreased significantly in *Lrp2*^RPE^ KO mice, resulting in choriocapillaris degeneration, which is suspected to control the physiological growth of the AL. Furthermore, *Vegf*^RPE^ KO mice demonstrated pathological elongation of AL, whereas *Vhl*^RPE^ KO mice, which have a higher level of *Vegf*, had shorter AL. Consequently, the level of VEGF released from RPE cells may be related to the AL. Nevertheless, another research group demonstrated that *Vegf*^RPE^ KO mice had small eyeballs (47). This appears to be attributable to the fact that the RPE-specific KO Cre genes used are markedly different. Another research group used the *Trp1*-Cre gene to knock out *Vegf* in RPE cells, and it has been reported that Cre expression begins around embryonic day 10.5 (48). Moreover, studies have reported that RPE-specific KO studies that used *Trp1*-Cre mice may have been a combination of the targeted loxP gene excision and/or the Cre transgene presence toxicity itself (49). The *Best1*-Cre gene, which we used in our study, begins to express itself around postnatal day 10. The choroidal endothelium adjacent to the RPE cells expresses high levels of Indian Hedgehog (50), a Hedgehog signaling pathway protein that is required for proper cell differentiation, and VEGF secreted by RPE cells is important in the development and maintenance of the choriocapillaris. Consequently, a VEGF shortage during the embryonic period causes choriocapillaris dysplasia, which interferes with normal cell development and causes microphthalmia. After birth, a physiological amount of VEGF is still required to maintain the choriocapillaris layer and regulate proper AL growth.

Our study also revealed a significant thinning of retinal thickness, decrease of retinal vessels and atrophy and dysfunction of photoreceptors in *Lrp2*^RPE^ KO mice, which were all consistent to the clinical features of pathological myopia complications (1). In addition, our previous study also showed that conditional knock out of *Vegf* from RPE cells lead to vision loss due to the damage of photoreceptors (24). Therefore, we speculated that all these changes in retinal layers were secondary change to the extremely ocular enlargement.

In conclusion, physiological secretion of VEGF is required for the development and maintenance of the choriocapillaris, which is required for the proper development of AL. Our hypothesis might only partially explain why AL increased in patients with myopia, as we cannot fully elucidate why *Lrp2*^RPE^ KO mice have such a dramatic phenotype, and more research is needed to determine the actual mechanism of myopia onset and progression. However, we believe that the RPE, which is the main source of VEGF, and choriocapillaris, could be promising targeted tissues for myopia control studies.

## Materials and methods

### Mice

All procedures were approved by the Ethics Committee on Animal Research of the Keio University School of Medicine adhered to the Association for Research in Vision and Ophthalmology Statement for the Use of Animals in Ophthalmic and Vision Research, the Institutional Guidelines on Animal Experimentation at Keio University, and the Animal Research: Reporting of In Vivo Experimental guidelines for the use of animals in research. All wild-type C57BL/6 J mice were obtained from CLEA Japan Inc. The *Lrp2*-flox mouse line containing loxP flanked exon 72 to 74 was generated (TransGenic Inc.). *Best1*-Cre mice (51), *Chx10*-Cre (52), and *Vhl-*flox (53) mice were obtained from the Jackson Laboratory. *Vegf*-flox (54) mice were kindly provided by Genetech. Mice with homozygous conditional inactivation of the *Lrp2* gene in RPE cells were generated by breeding *Best1*-Cre transgenic mice with mice bearing a loxP-flanked *Lrp2* allele to create first generation *Best1*-Cre, *Lrp2* heterozygous mice (*Lrp2*^floxed/–^) on a C57BL/6 background. Heterozygous *Best1*-Cre, *Lrp2* (C57BL/6) were subsequently crossed with mice bearing loxP-flanked *Lrp2* alleles to obtain homozygous *Best1*-Cre, *Lrp2* mice (*Lrp2*^floxed/floxed^). Mice with homozygous conditional inactivation of the *Vhl* and *Vegf* gene in RPE cells, conditional inactivation of the *Lrp2* gene in neural retina were generated by the same procedures. Cre-negative littermates served as controls in all experiments. Mice were genotyped by PCR analysis with tail DNA. GoTaq DNA polymerase and reagents (Promega, USA) were used to amplify the products. The PCR conditions were as follows: initial denaturation (95°C for 2 min), followed by 13 cycles of denaturation (95°C for 15 s), annealing (68°C for 15 s) and extension (72°C for 1 min), followed by 22 cycles of denaturation (95°C for 15 s), annealing (60°C for 15 s) and extension (72°C for 45 s) and a final extension at 72°C for 5 min. The PCR products were separated on a 2.0% agarose gel. The primers used were as follows: *Lrp2* (forward: GCCTTGATGTGGTCTATTGT, reverse: GATATCCTTACTTGATGAGGACCCTCC); *Vegf* (forward: CCTGGCCCTCAAGTACACCTT, reverse: TCCGTACGACGCATTTCTAG); *Vhl* (forward: CTAGGCACCGAGCTTAGAGGTTTGCG, reverse: CTGACTTCCACTGATGATGCTTGTCACAG); *Best1*-Cre (forward: ATGCGCCCAAGAAGAAGAGGAAGGTCTCC, reverse: TGGCCCAAATGTTGCTGGATAGTTTTTA); *Chx10*-Cre (forward: GAGGAAGGCCCCATATTTGT, reverse: AGGCAAATTTTGGTGTACGC). Floxed mice without the Cre transgene were used as control littermates.

### Refraction, axial length, and choroidal thickness measurements

Refractions and ALs were measured as described in the previous studies (30). In brief, the refractive state was measured using an infrared photorefractor (Steinbeis Transfer Center). To assure mydriasis and cycloplegia, tropicamide and phenylephrine hydrochloride solution (Mydrin-Pophthalmic solution, Santen Pharmaceutical) was applied to the mouse eye 5 min before the measurement. The general anesthesia was induced by a combination of midazolam (Sandoz K.K.), medetomidine (Domitor, Orion Corporation), and butorphanol tartrate (Meiji Seika Pharma Co., Ltd.), or MMB. Refraction was measured along the optic axis. Following the refraction measurement, a spectral domain-OCT system (Envisu R4310, Leica) tuned for mice was used to examine the AL and choroidal thickness. AL is the distance between the vertex of the cornea and the RPE layer near the optic nerve. According to a previous study, choroidal thickness was measured using the OCT system (55). In brief, the area of the circumference (0.5 mm) from the disk circled at the border of the RPE, and the posterior surface of the choroid was quantified using ImageJ (NIH) (Supplementary Material, Fig. S9). The average choroidal thickness was calculated by dividing the circumference by the area. Measurements of refraction, AL, and choroid thickness were performed once a week from 3 to 10 weeks and once in 2 weeks from 10 to 16 weeks of age in C57BL/6 J, *Vhl*^floxed/floxed^, and *Vegf*^loxed/floxed^  *Best1*-Cre recombinase mice. Measurements of AL were performed at 3 weeks of age, once every 2 weeks from 4 to 28 weeks of age, and once every 6 weeks from 28 to 40 weeks of age in *Lrp2*^floxed/floxed^  *Best1*-Cre recombinase mice. Using the same OCT system, measurements of corneal thickness, anterior chamber depth, lens thickness, and vitreous chamber depth plus retinal thickness were performed in 8-week-old *Lrp2*^floxed/floxed^  *Best1*-Cre and *Chx10*-Cre recombinase mice.

### Lens-induced myopia model

According to previous reports (30), MMB administered general anesthesia to mice. The scalp was dissected to expose an area of the skull measuring 0.8 cm^2^, and the periosteum was removed with etching fluid. Subsequently, a pair of eyeglasses was attached to the mouse's head using a self-cure dental adhesive system (Super-Bond, SUN MEDICAL). The eyeglasses were prepared specifically for the mice using a three-dimensional printer. The eyeglasses had a joint part that permitted the left and right frame positions to be adjusted to fit the shape of a mouse's skull or be removed for cleaning. The lenses on the eyeglasses were customized from human hard contact lenses by a manufacturer in Japan. As an internal control, all the left sides of the eyeglasses used in this study were attached with 0 D lenses, and the right sides were attached with −30 D lenses. Each mouse's eyeglasses were removed for cleaning at least twice per week.

### Immunohistochemistry

For flat-mount immunostaining of the retina/RPE/choroid, mice were deeply anesthetized using isoflurane inhalation and euthanized by cervical dislocation. The eyes were then removed and fixed for 45 min at room temperature in 4% paraformaldehyde and washed for 20 min with PBS containing 0.5% Triton X-100 (PBST) three times, followed by blocking in 10% normal donkey serum (blocking buffer) for 1 h. Primary antibodies were applied in blocking buffer at 4°C for two overnights, washed three times in PBST (20 min per wash), followed by incubation with secondary antibodies. Finally, the retina/RPE/choroid was washed three times in PBST (20 min per wash) and mounted in Fluoro-Gel (Electron Microscopy Sciences). For cross section immunostaining, eyes were enucleated and fixed by immersion in SUPERFIX fixation solution (Kurabo Industries; Osaka, Japan) 3 days prior to dehydration and paraffin embedding. 5 µm paraffin sections were cut on a microtome (Yamato Kohki REM-710; Saitama, Japan). Sections were heated in a pressure cooker with citrate buffer for antigen retrieval for 5 min after deparaffiniation. Washed the sections for 5 min with PBS three times, followed by blocking in 10% normal donkey serum (blocking buffer) for 1 h. Primary antibodies were applied in blocking buffer at 4°C for 1 overnight, washed three times in PBS (5 min per wash), followed by incubation with secondary antibodies. Finally, the sections were washed three times in PBS (5 min per wash) and mounted in Fluoro-Gel (Electron Microscopy Sciences). The primary antibodies and dilutions used were as follows: rat anti-endomucin (1:500; Millipore MAB2624), isolectin B4-488 (1:1000; ThermoFisher Scientific I21411), isolectin B4-594 (1:1000; ThermoFisher Scientific I21413), rat anti-ZO-1 (1:125; Invitrogen 14,977,682), rabbit anti-Lrp2 (1:1000; Abcam ab76969), Rabbit Collagen I antibody (1:500; Novus NB600-408). Images were obtained using a confocal microscope (LSM710; Carl Zeiss; Oberkochen, Germany).

### Retinal pigment epithelium isolation

Primary mouse RPE cells were prepared following protocol (56). The 8-week-old *Lrp2*^floxed/floxed^  *Best1*-Cre recombinase mice and control mice were sacrificed by decapitation. Eyes were removed and washed in HBSS without Ca^2+^ or Mg^2+^ using 10 mM HEPES. Three incisions were made in the cornea, and the lens was removed completely. The eye was placed in a HEPES-free HBSS buffer. The eyes were incubated in hyaluronidase solution at 37°C for 45 min in a 5% carbon dioxide aerated incubator to detach the neural retina from the RPE. Each eye was placed in a new well with 1.5 mL of cold HBSS HEPES buffer per well and incubated on ice for 30 min. After washing, each eye was placed in a 35-mm culture dish containing fresh HBSS HEPES buffer. The iris epithelium and cornea were removed, and the retina was pulled away. Each eye cup was transferred to a different 12-well plate containing fresh trypsin-EDTA (1.5 mL) per well and incubated the eyecups at 37°C for 45 min in a 5% carbon dioxide incubator. Each eye cup was held by the optic nerve and shaken face down into a 12-well plate containing 1.5 mL of 20% FBS in HBSS HEPES buffer until a complete detachment of the RPE sheets were achieved. RPE cells from the four eyes of two mice were used for each sample.

### Real-time PCR analysis

TRI reagent (MRC Global, Cincinnati, OH, USA) was used to dissolve total RNA from RPE cells, which were then transferred to Econospin columns for RNA extraction and collection (GeneDesign, Osaka, Japan). The RNA samples were analyzed using an ND-2000 spectrophotometer (Thermo Fisher Scientific, DE, USA) to evaluate the quantity and quality of the samples after washing with buffer RWT and RPE (Qiagen, Hilden, Germany). The PrimeScript II First Strand cDNA Synthesis Kit (Takara Bio, Otsu, Japan) was used for reverse transcription. THUNDERBIRD SYBR qPCR Mix (TOYOBO, Osaka, Japan) and a QuantStudio 12 K Flex Real-Time PCR machine (Applied Biosystems, Waltham, MA, USA) were used for real-time PCR. The primers used were as follows: mouse *Gapdh* (forward: AGGAGCGAGACCCCACTAAC, reverse: GATGACCCTTTTGGCTCCC); mouse *Vegf* (forward: CTCCAGGGCTTCATCGTTA, reverse: CAGAAGGAGAGCAGAAGTCC); mouse *Lrp2* (forward: GGAGGAACCAATCTGTTGTAATGT, reverse: GATGGTTGCCTGGAGGG). The fold change between the levels of different transcripts was calculated using the ΔΔCT method.

### Electroretinography

A Ganzfeld dome, an acquisition system (PuREC, MAYO Corporation, Japan), and LED stimulators were used to acquire scotopic and photopic ERGs. The mice were anesthetized with MMB under dim red light after overnight dark adaptation. Tropicamide and phenylephrine hydrochloride (Santen Pharmaceutical Co., Ltd., Japan) was used to dilate the pupils. The active electrodes were recorded with contact electrodes, and the reference electrode was placed under the scalp. A clipping electrode to the tail served as the ground. ERG responses were obtained from both the eyes of each animal. Rod responses were recorded in the dark with a stimulus intensity of 0.02 cd/m^2^, and four responses were recorded and averaged automatically. A white flash of 50 cd/m^2^ was used to evoke mixed rod and cone responses (mixed ERGs). The mice were adapted for 5 min under a white background (30 cd/m^2^) to assess photopic ERGs. Cone responses were recorded with a 20 cd/m^2^ stimulus, and the average of 32 individual responses was calculated. The low pass of the amplifiers was set to 30 Hz.

### Electron microscopy analysis

Eyes were enucleated and fixed with 2.5% glutaraldehyde in PBS overnight at 4°C and then rinsed in 0.1 M sodium cacodylate buffer for 1 h. The eye cups were then postfixed in 1% OsO4 in 0.1 M cacodylate buffer for 2 h, followed by another 1-h wash and dehydration with graded ethanol solutions. Samples were incubated overnight in a 1:2 mixture of propylene oxide and Epon/Araldite (Sigma-Aldrich) and then placed in 100% resin, followed by embedding. The blocks were sectioned and observed using a JEM1400 plus transmission electron microscope (JEOL, Tokyo, Japan) at an acceleration voltage of 100 kV.

### Analysis of images

For immunohistochemical analyses, choroidal vascular density and quantification of *Lrp2* expression in RPE were analyzed using ImageJ software (NIH), as described previously (25). Retinal vascular density was quantified using the IMARIS software (version 9.7; Oxford Instruments, UK) according to the manufacturer's instructions. For general histological examination, sections were counterstained with H&E, and images were acquired using an OlympusBX53 microscope (Olympus, Tokyo, Japan). The optic nerve divides the entire eyeball into two hemispheres. The complete length of each optic nerve to the ciliary body was measured using the OlympusBX53 microscope, and the total length was divided into five equal portions, and the choroidal thickness corresponding to each spot was measured in P1, and 1-, 2-, 3-, 6-, 8-, and 10-week-old C57BL/6 J mice. The choroidal thickness of 10 spots was measured in each section, and the average was calculated for each time point. Using the same method, retinal, INL, ONL, choroidal, and scleral thickness were measured in 3-, 6-, and 8-week-old *Lrp2*^floxed/floxed^  *Best1*-Cre recombinase and control mice.

### Clinical study

The study was conducted with the approval of the Keio University School of Medicine Ethics Committee (No. 20,180,189). All procedures involving human subjects were performed in accordance with the Declaration of Helsinki. The data of 65 to 75-year-old patients who visited the outpatient department of Keio University Hospital from 2019 to 2021, with an eye axis length of 26.5 mm or more, IOL-Master (IOLMaster 500, Zeiss, Jena, Germany), and OCT (RS-3000, Nidek, Aichi, Japan) examination results, and follow-up for more than 6 months, were retrospectively collected in this study. Basic characteristic, including as VA, IOP, and SE, are also shown in Supplementary Material, Table S15.

### Statics

All data were presented as the means ± SD. A two-tailed Student's *t*-test (Excel; Microsoft) was used to compare the mean variables of the two groups. When three or more groups were compared, data were analyzed using analysis of variance followed by the Tukey–Kramer multiple-comparison test (GraphPad Prism 9.0). Statistical significance was set at *P* < 0.05.

## Supplementary Material

pgac166_Supplemental_File

## Data Availability

All study data are included in the article and/or supporting information.
