# Correction to: Vascular endothelial growth factor from retinal pigment epithelium is essential in choriocapillaris and axial length maintenance

**DOI:** 10.1093/pnasnexus/pgad467

**Published:** 2024-01-30

This is a correction to: Yan Zhang, Heonuk Jeong, Kiwako Mori, Shin-Ichi Ikeda, Chiho Shoda, Yukihiro Miwa, Ayaka Nakai, Junhan Chen, Ziyan Ma, Xiaoyan Jiang, Hidemasa Torii, Yoshiaki Kubota, Kazuno Negishi, Toshihide Kurihara, Kazuo Tsubota, Vascular endothelial growth factor from retinal pigment epithelium is essential in choriocapillaris and axial length maintenance, *PNAS Nexus*, Volume 1, Issue 4, September 2022, pgac166, https://doi.org/10.1093/pnasnexus/pgac166

During a retroactive audit conducted by *PNAS Nexus*, it was discovered that this paper was missing a statement acknowledging compliance with the *PNAS Nexus* Human and Animal Participants and Clinical Trials policy:

Informed consent was obtained from all participants involved in the research by an opt-out method.

This error has been corrected in the original article.

