# Supplementary material for: Vascular endothelial growth factor from retinal pigment epithelium is essential in choriocapillaris and axial length maintenance
Source: PNAS Nexus. 2022 Aug 24;1(4):pgac166. doi: 10.1093/pnasnexus/pgac166 (PMC9802415; doi:10.1093/pnasnexus/pgac166)
Supplement: pgac166_Supplemental_File [file pgac166_supplemental_file.docx]

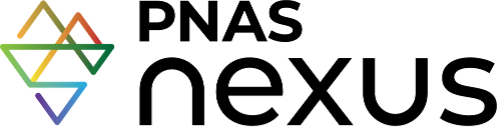


**Supplementary Information for**

Vascular Endothelial Growth Factor from Retinal Pigment Epithelium is Essential in Choriocapillaris and Axial Length Maintenance

Yan Zhang, Heonuk Jeong, Kiwako Mori, Shin-Ichi Ikeda, Chiho Shoda, Yukihiro Miwa, Ayaka Nakai, Junhan Chen, Ziyan Ma, Xiaoyan Jiang, Hidemasa Torii, Yoshiaki Kubota, Kazuno Negishi, Toshihide Kurihara, Kazuo Tsubota

Toshihide Kurihara, MD, PhD

Laboratory of Photobiology, Department of Ophthalmology, Keio University School of Medicine; 35 Shinanomachi, Shinjuku-ku, Tokyo 160-8582, Japan

Tel: +81-3-5363-3204, Fax: +81-3- 5363-3274

E-mail: [kurihara@z8.keio.jp](mailto:kurihara@z8.keio.jp)

Kazuo Tsubota, MD, PhD

Tsubota Laboratory, Inc., 34 Shinanomachi, 304 Toshin Shinanomachi Ekimae Building, Shinju-ku, Tokyo 160-0016, Japan.

Tel: +81-3-6384-2866

E-mail:  [tsubota@tsubota-lab.com](mailto:tsubota@tsubota-lab.com)

**This PDF file includes:**

Figures S1 to S9

Tables S1 to S33


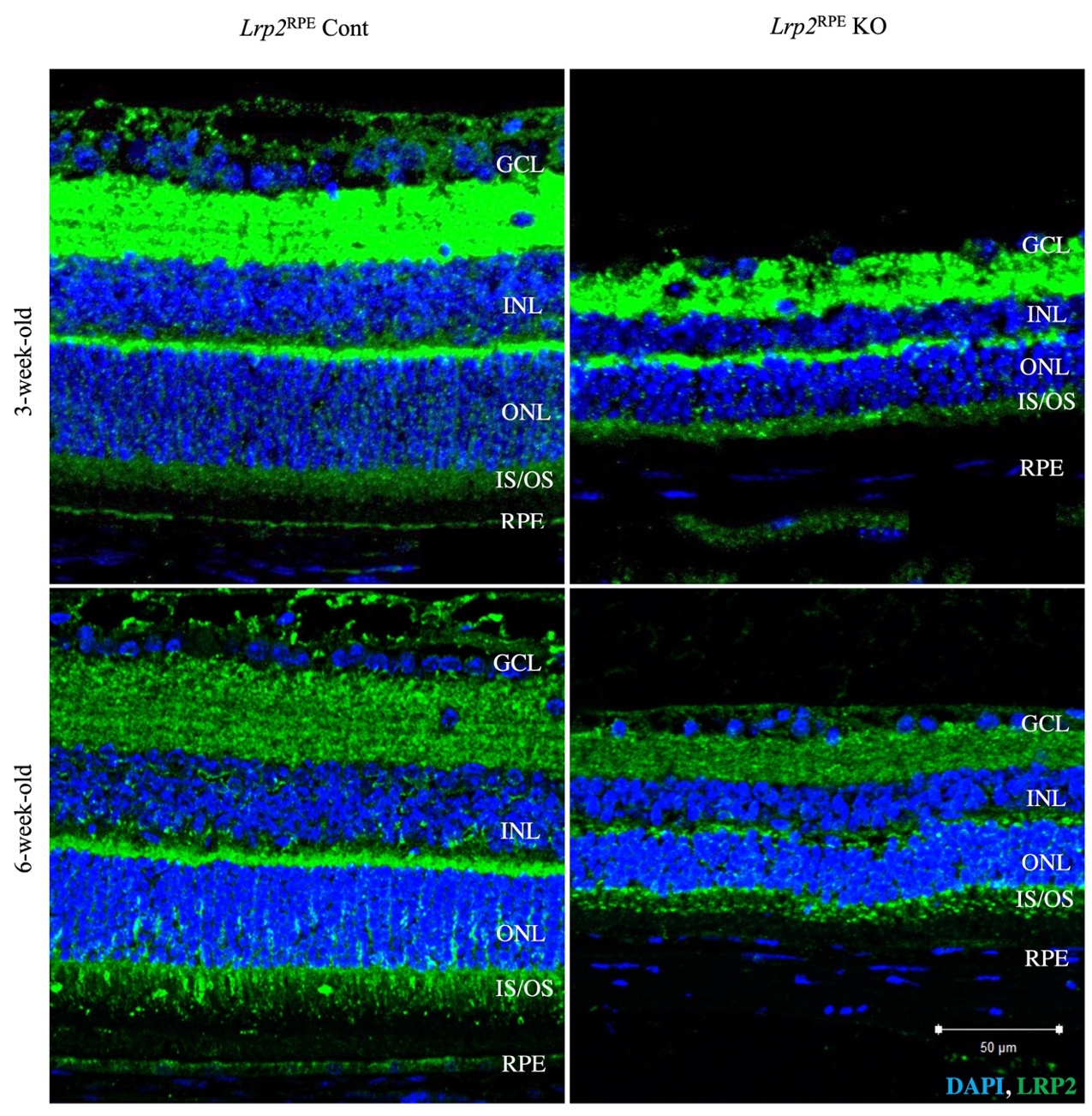


**Fig. S1. Immunohistochemical localization of LRP2 in *Lrp2*^RPE^ KO (right panel) and control mice (left panel).** LRP2 is shown in green and cell nuclei in blue. LRP2 were found in neural retina and RPE cells in control mouse while *Lrp2*^RPE^ KO mouse showed disappearance of LRP2 in RPE cells. GCL ganglion cell layer, INL inner nuclear layer, ONL outer nuclear layer, IS/OS inner segment/outer segment, RPE retinal pigment epithelium. Scale bars 50 μm.


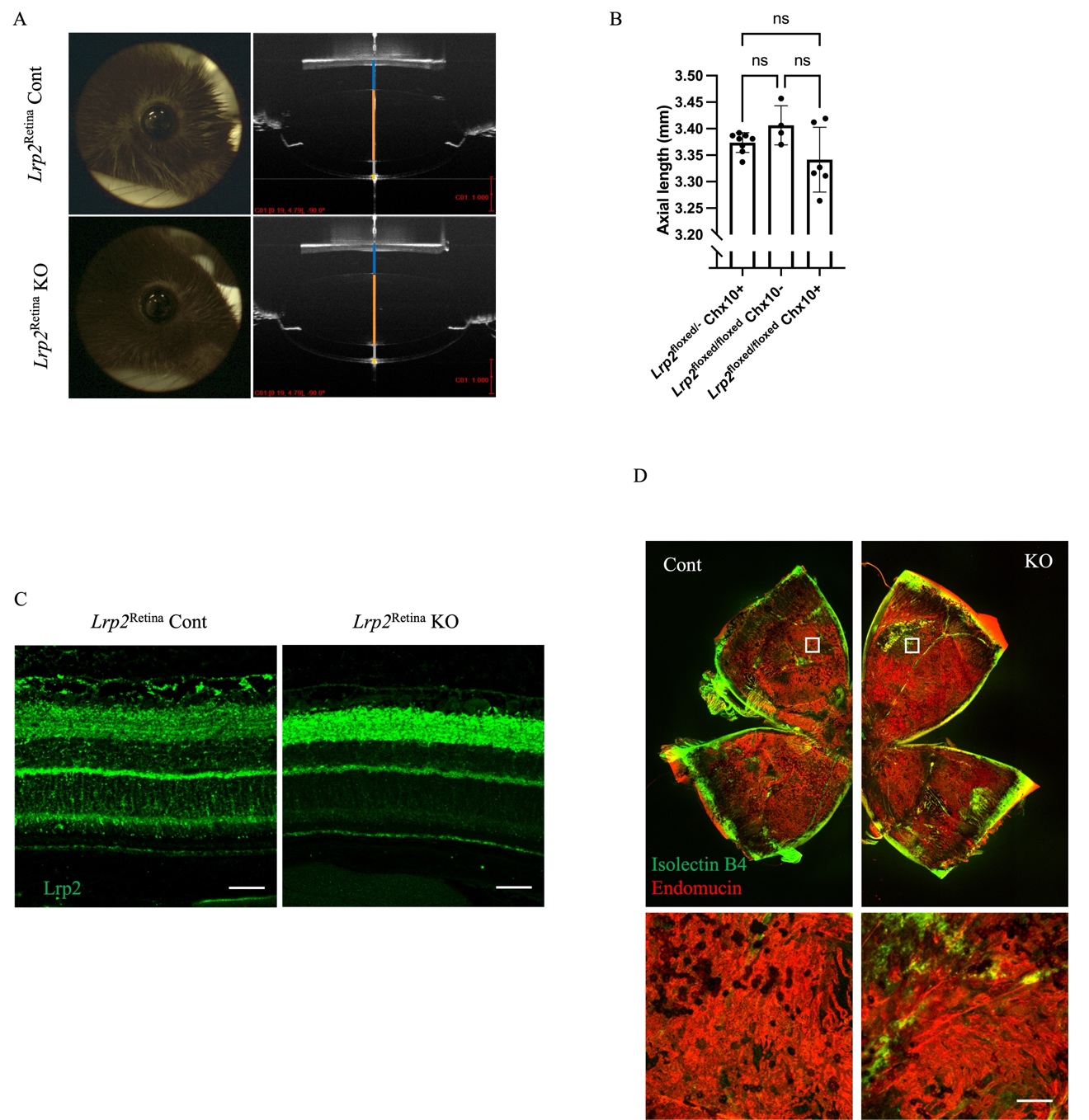


**Fig. S2. *Lrp2*^Retina^ KO mice showed no significant differences with *Lrp2*^Retina^ Control mice.** (A) Representative OCT image of the whole eye in 8-week-old *Lrp2*^Retina^ Cont and *Lrp2*^Retina^ KO mouse (vitreous chamber depth + retinal thickness; blue line, lens thickness; orange line, anterior chamber depth; gray line, corneal thickness; yellow line). Scale bar in red: 1 mm. (B) AL of *Lrp2*^floxed/-^ *Chx10*-cre+ mice showed no significant difference with *Lrp2*^floxed/floxed^ *Chx10*-cre- (*Lrp2*^Retina^ control) and *Lrp2*^floxed/floxed^ *Chx10*-cre+ (*Lrp2*^Retina^ KO) mice. ns: no significance. One-way ANOVA tests. (C) Immunohistochemical localization of LRP2 in *Lrp2*^Retina^ KO (right panel) and control mice (left panel). LRP2 is shown in green. *Lrp2*^Retina^ KO mouse showed lower signals in neural retina. Scale bar: 20μm. (D) Representative immunohistochemistry of choroid flat-mount (green: isolectin B4, red: endomucin) of 10-week-old *Lrp2*^Retina^ Cont and *Lrp2*^Retina^ KO. Scale bar: 1 mm (upper panels), 20 μm (lower panels).


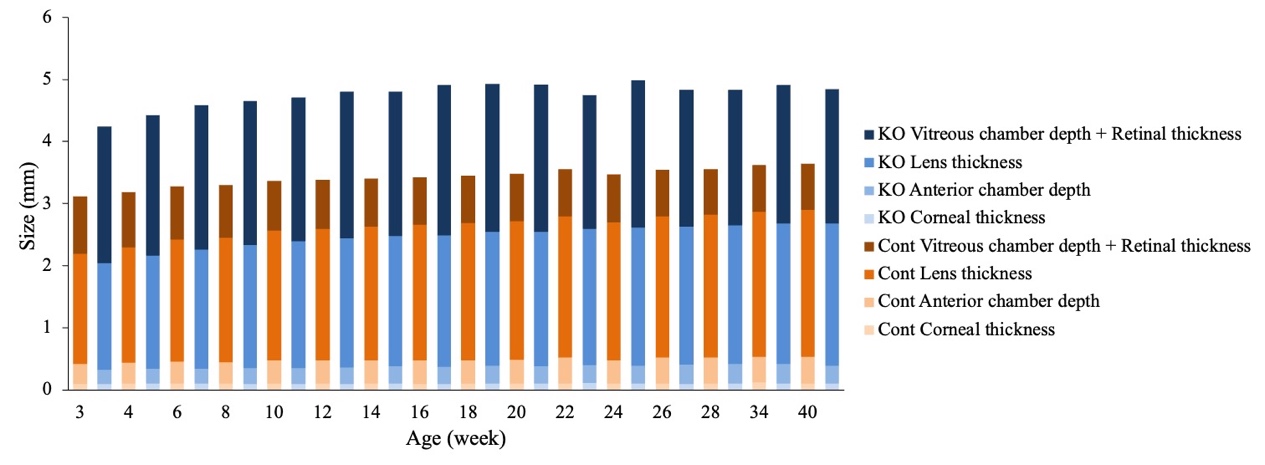


**Fig. S3. Development of eye sizes in *Lrp2*^RPE^ KO and control mice from 3 weeks to 40 weeks old.** Development of corneal thickness, anterior chamber depth, lens thickness, vitreous chamber depth and retinal thickness in *Lrp2*^RPE^ KO and control mice from 3 weeks to 40 weeks old (n=3-8), *Lrp2*^RPE^ KO showed a longer total thickness of eye sizes from the beginning.


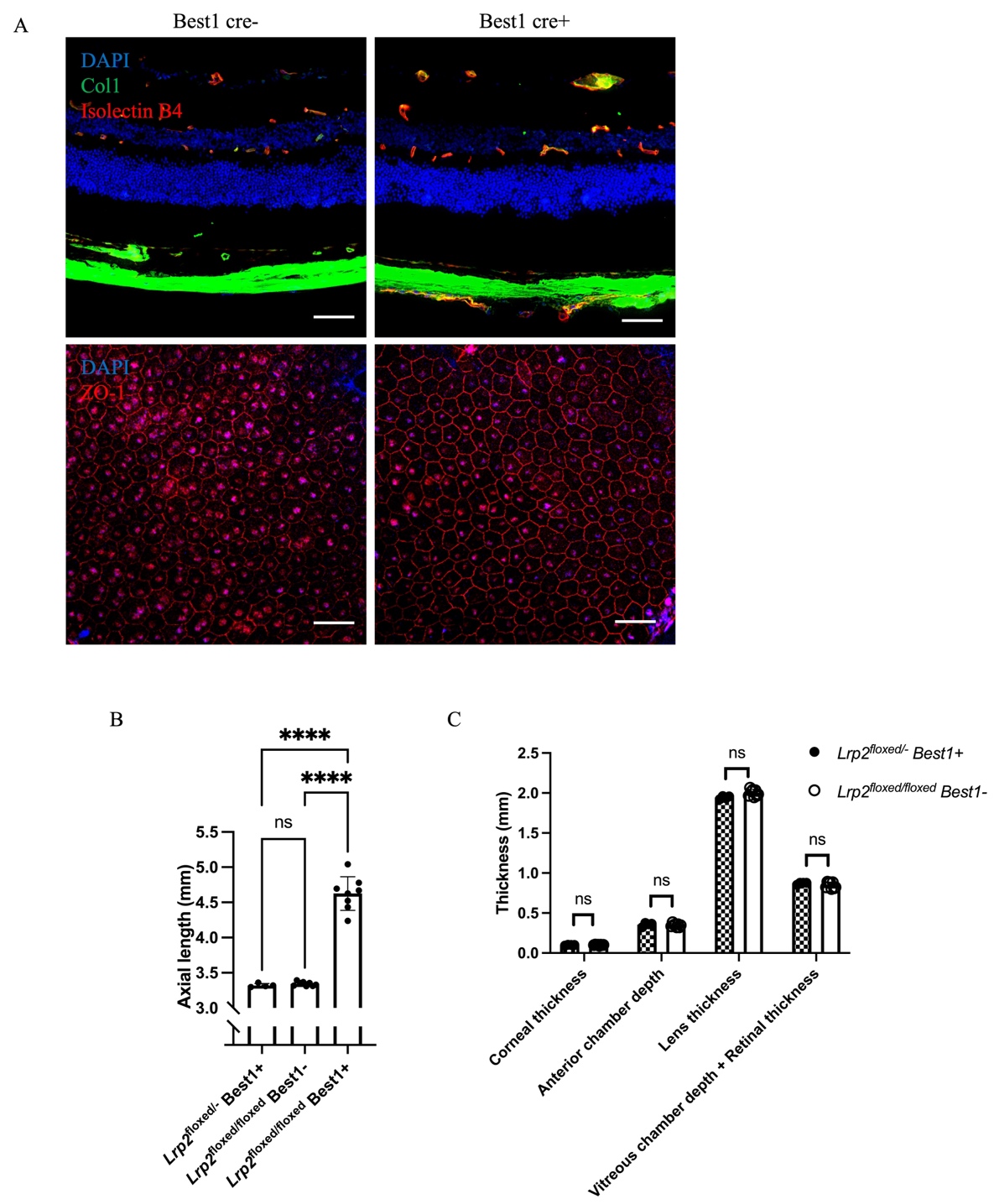


**Fig. S4.** ***Best1*-cre itself does not alter the experimental outcomes.** Immunohistochemical staining showed no obvious difference between *Best1*-cre+ mice and *Best1*-cre- mice in cross sections (A, upper panels; Col1 is shown in green, cell nuclei in blue and vessels in red) and flat-mount (A, lower panels; cell nuclei in blue and tight junctions of RPE cells are shown in red). Scale bar: 50 μm. AL of *Lrp2*^floxed/-^ *Best1*-cre+ mice showed no significant difference with *Lrp2*^floxed/floxed^ *Best1*-cre- (*Lrp2*^RPE^ control) mice. Only *Lrp2*^floxed/floxed^ *Best1*-cre+ (*Lrp2*^RPE^ KO) revealed significant elongation in AL (B). No significant differences are shown in other ocular parameters Between *Lrp2*^floxed/-^ *Best1*-cre+ mice and with *Lrp2*^floxed/floxed^ *Best1*-cre- (*Lrp2*^RPE^ control) mice (C). ns: no significance, ****P < 0.0001, one-way ANOVA tests (B), 2-tailed Student’s t tests (C). Error bars indicate mean ± SD.


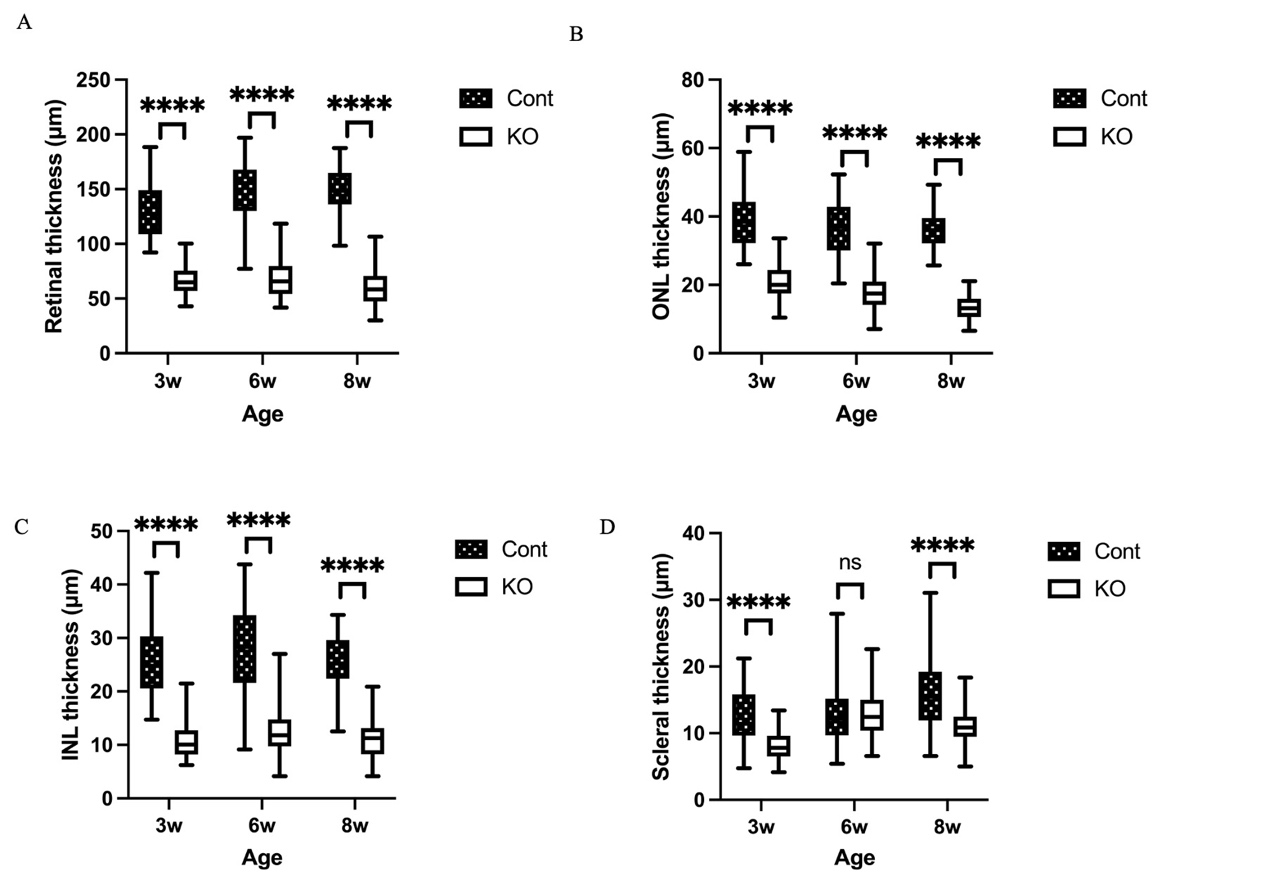


**Fig. S5. *Lrp2*^RPE^ KO mice showed thinner total retinal, INL, ONL and scleral thickness.** H&E staining of the paraffin sections revealed that thickness of the whole retinal layer, ONL layer and INL layer and scleral layer (A-D) were significantly decreased in the mutant eyes from 3 to 8 weeks old (n=3-6). ***P < 0.001, 2-tailed Student’s t tests (A-D). Error bars indicate mean ± SD

**
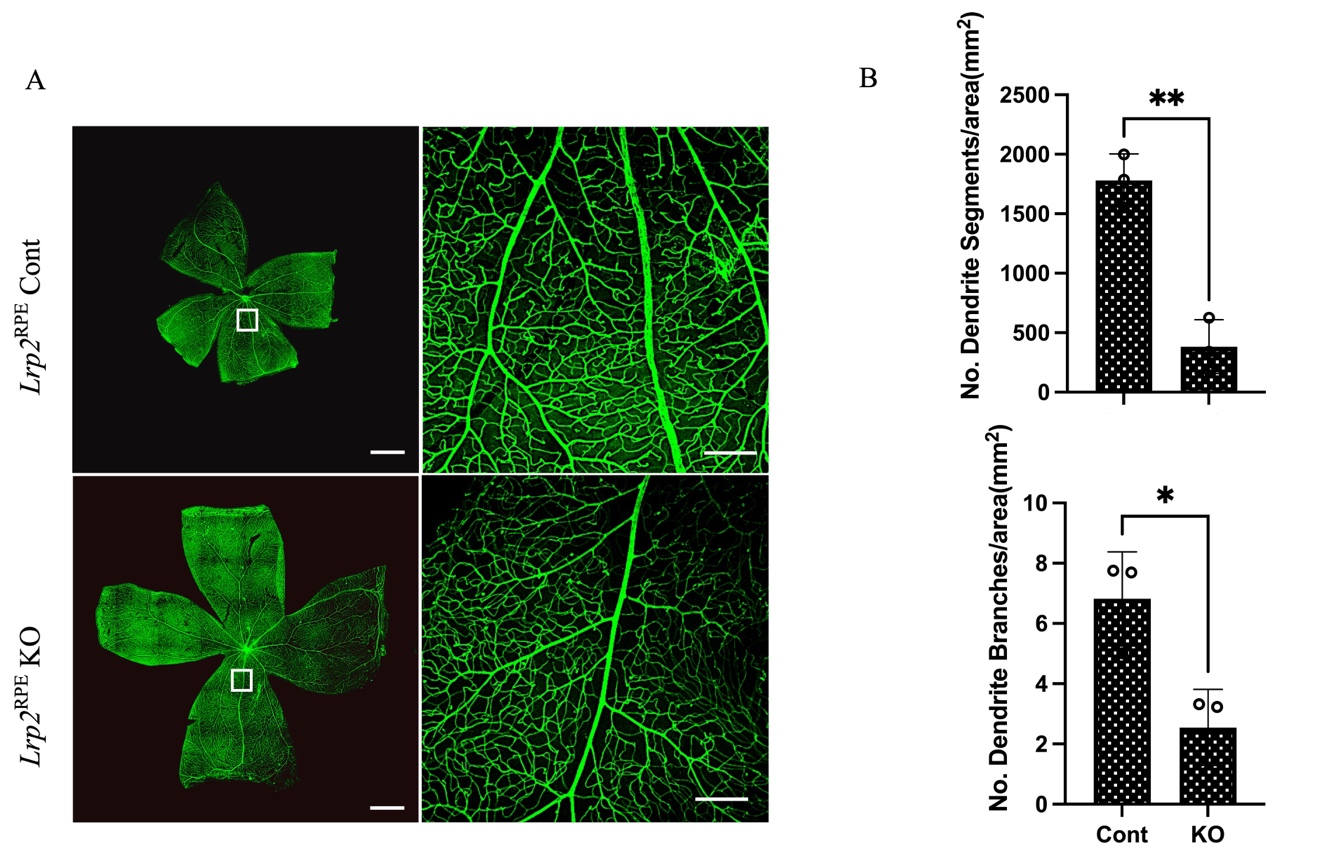
 Fig. S6. *Lrp2*^RPE^ KO mice had a decreased retinal vascular density.** Retina flat-mount immunohistochemistry are shown in (A), which reveals decreased vascular density in *Lrp2*^RPE^ KO mice (n=3) (B). Scale bar: 1 mm (A). *P<0.05, **P < 0.01, 2-tailed Student’s t tests (B). Error bars indicate mean ± SD.


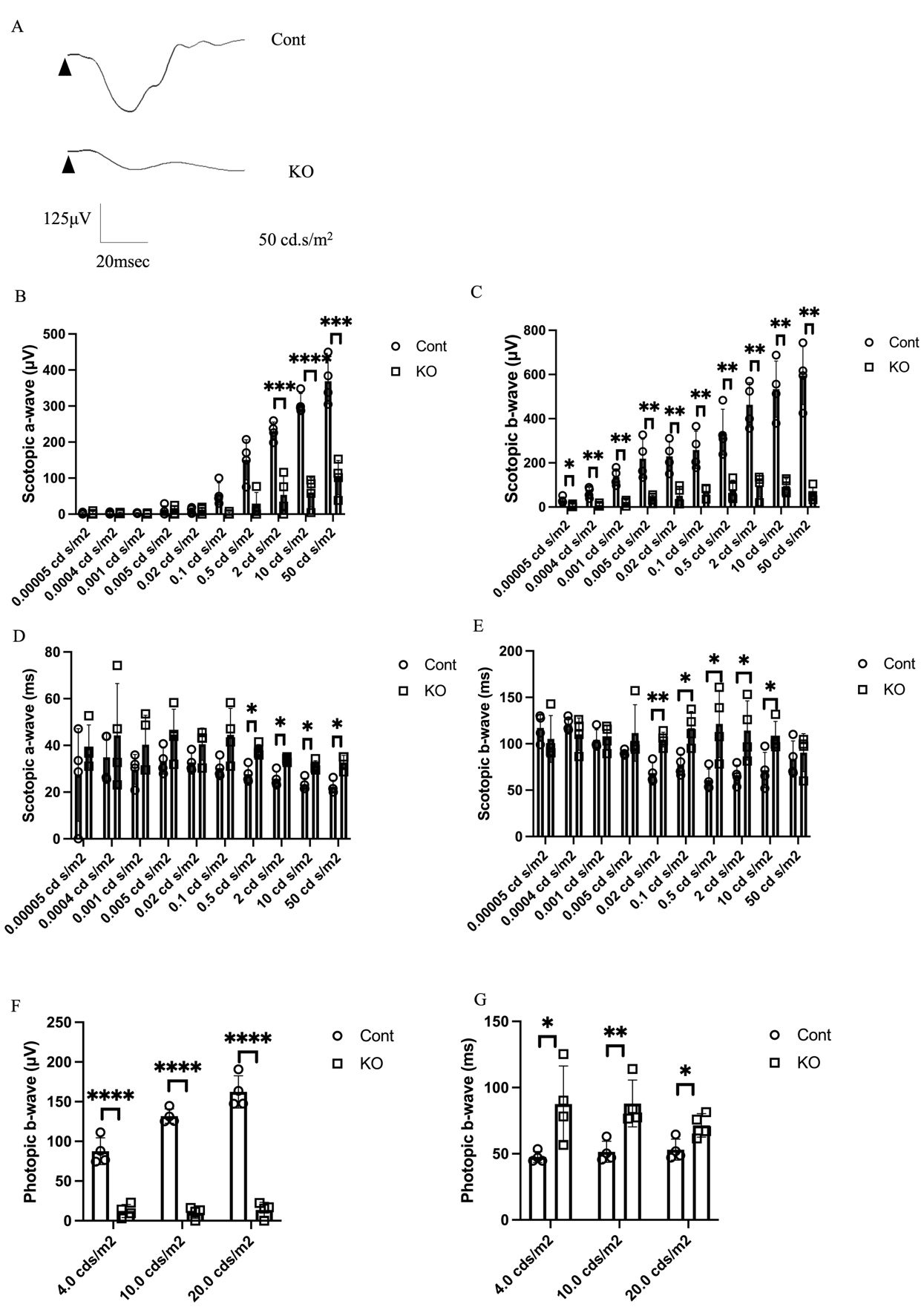


**Fig. S7. *Lrp2*^RPE^ KO mice demonstrated damaged vision function.** Full-field ERGs performed on *Lrp2*^RPE^ KO and control mice at 6 weeks old. ERG analyses reveal that damaged photoreceptor function is observed in *Lrp2* mutant mice (n=4) (A-G). *P < 0.05, **P < 0.01, ***P < 0.001, ****P < 0.0001, 2-tailed Student’s t tests (B-G). Error bars indicate mean ± SD.


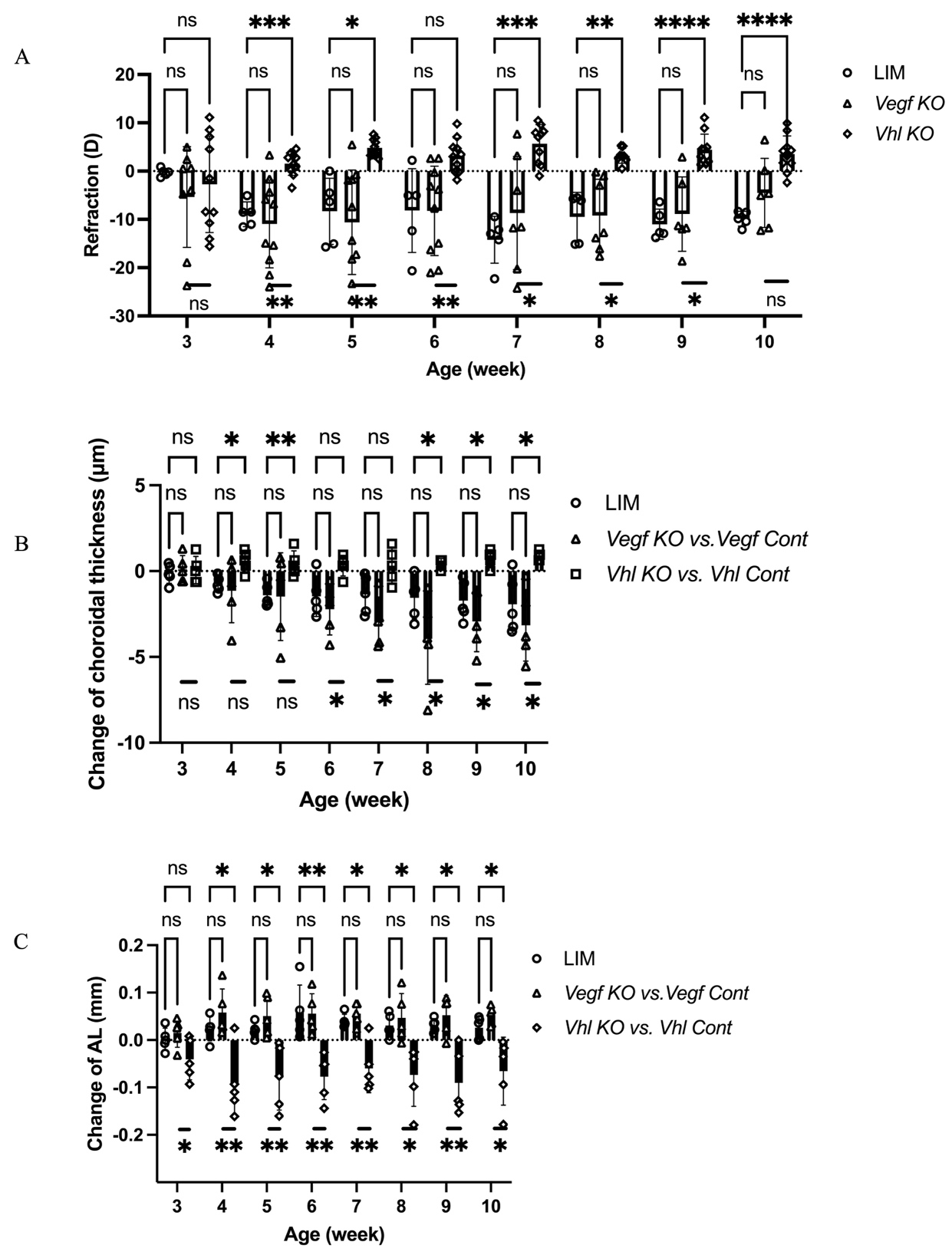


**Fig. S8.** ***Vegf*^RPE^ KO mice showed similar myopic feactures as LIM mice.** (A) No significant difference was observed in refraction status between LIM and *Vegf*^RPE^ KO mice. (B and C) Change comparison in choroidal thickness (I) and axial length (J) of LIM, *Vegf*^RPE^ KO, and *Vhl*^RPE^ KO mice showing no significant difference between LIM and *Vegf*^RPE^ KO mice. *p<0.05, **p < 0.01, ***p<0.001, ****p < 0.0001, one-way repeated measures of ANOVA. Graphs represent mean ± SD.


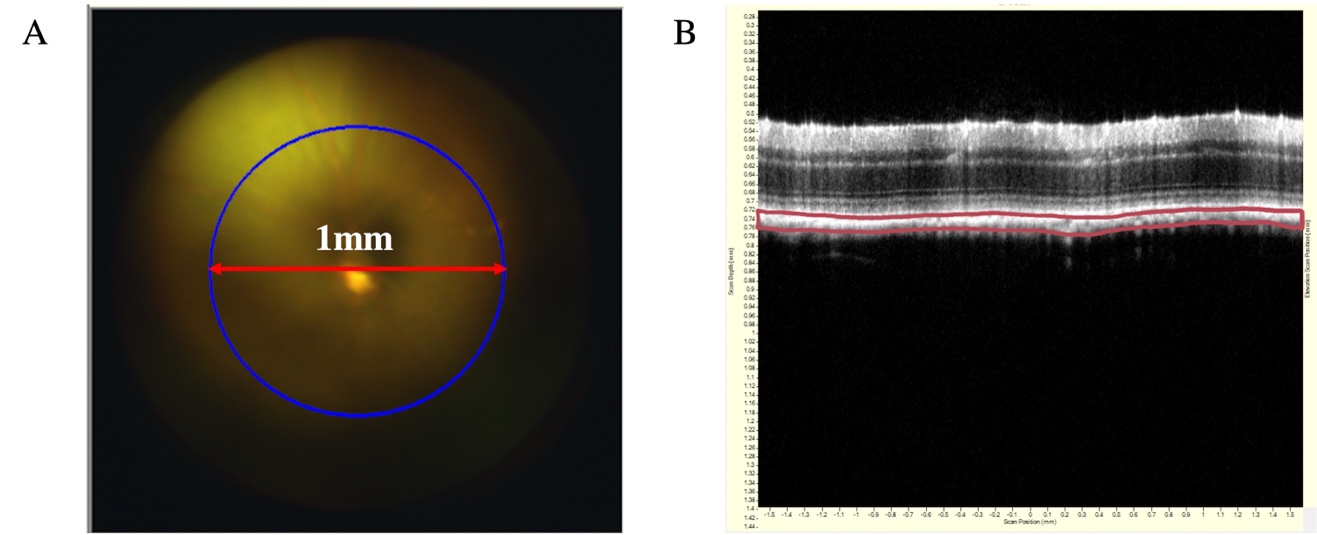


**Fig. S9. Quantification of choroidal thickness utilizing the SD-OCT system.** A show a fundus photography captured in the OCT system. The blue circle indicates the circumference at 0.5 mm from the disc. B shows a cross sectional image of the circumference captured in the OCT system.

The area of the circumference at 0.5 mm from the disc circled at the border of the retinal pigment epithelium and the posterior surface of the choroid was quantified by ImageJ.

| Table S1: Numeric data for *LRP2* relative area: Figure 1B | | |
| --- | --- | --- |
| 2-tails Student’s t test | | |
| Types of mice: *Lrp2*^RPE^ | | **p-values: comparison between:** |
| Control | KO | Control and KO |
| Min/*P*_25_/Median/ *P*_75_/Max | Min/*P*_25_/Median/ *P*_75_/Max |  |
| 76.768/86.295/89.359/91.456/97.372 | 3.400/7.161/11.102/18.944/26.620 | **<0.0001** |
| Min: minimum. *P*_25_: 25^th^ percentile. *P*_75_: 75^th^ percentile. Max: Maximum. | | |

| Table S2: Numeric data for ocular parameters difference: Figure 1D | | | |
| --- | --- | --- | --- |
| 2-tails Student’s t test | | | |
| Ocular parameters | **Types of mice: *Lrp2*^RPE^** | | **p-values: comparison between:** |
|  | Control | KO | Control and KO |
|  | Min/*P*_25_/Median/ *P*_75_/Max | Min/*P*_25_/Median/ *P*_75_/Max |  |
| Axial length | 3.310/3.313/3.343/3.365/3.392 | 4.237/4.455/4.648/4.758/5.042 | **<0.0001** |
| Corneal thickness | 0.091/0.099/0.100/0.103/0.105 | 0.093/0.095/0.099/0.103/0.104 | 0.765 |
| AC depth | 0.321/0.324/0.350/0.359/0.384 | 0.246/0.255/0.261/0.277/0.281 | **<0.0001** |
| Lens thickness | 1.947/1.965/2.003/2.039/2.066 | 1.953/1.974/2.001/2.028/2.038 | 0.864 |
| VC depth + retinal thickness | 0.802/0.812/0.850/0.884/0.890 | 1.775/2.042/2.203/2.336/2.675 | **<0.0001** |
| AC: anterior chamber. VC: vitreous chamber. Min: minimum. *P*_25_: 25^th^ percentile. *P*_75_: 75^th^ percentile. Max: Maximum. | | | |

| Table S3: Numeric data for axial length: Figure 1E | | | | | |
| --- | --- | --- | --- | --- | --- |
| One-way repeated measures ANOVA: F(3, 28) = 6.096, p=0.0025 | | | | | |
| Types of mice | Min/*P*_25_/Median/ *P*_75_/Max | **p-values: comparison between:** | | | |
|  |  | different types | | | |
| *Lrp2*^Retina^ Cont | 3.337/3.369/3.384/3.403/3.457 | with |  |  |  |
| *Lrp2*^Retina^ KO | 3.264/3.312/3.342/3.404/3.419 | 0.9241 | with |  |  |
| *Lrp2*^RPE^ Cont | 3.310/3.313/3.343/3.365/3.392 | 0.8899 | 0.9997 | with |  |
| *Lrp2*^RPE^ KO | 4.237/4.455/4.648/4.758/5.042 | **<0.001** | **<0.001** | **<0.001** | with |
| Min: minimum. *P*_25_: 25^th^ percentile. *P*_75_: 75^th^ percentile. Max: Maximum. | | | | | |

| Table S4: Numeric data for choroidal thickness: Figure 2B | | | |
| --- | --- | --- | --- |
| 2-tails Student’s t test | | | |
| Age(week) | **Types of mice**  ***Lrp2*^RPE^** | | **p-values: comparison between:** |
|  | Control | KO | Control and KO |
|  | Min/*P*_25_/Median/ *P*_75_/Max | Min/*P*_25_/Median/ *P*_75_/Max |  |
| 3 | 4.160/6.755/7.950/10.400/16.970 | 1.340/3.740/4.940/6.885/13.310 | **<0.0001** |
| 6 | 6.420/9.845/14.020/18.660/39.770 | 1.010/2.710/5.300/7.260/13.300 | **<0.0001** |
| 8 | 6.580/11.111/13.320/17.293/30.180 | 0.100/1.770/3.870/6.240/14.420 | **<0.0001** |
| Min: minimum. *P*_25_: 25^th^ percentile. *P*_75_: 75^th^ percentile. Max: Maximum. | | | |

| Table S5: Numeric data for vascular area: Figure 2D | | |
| --- | --- | --- |
| 2-tails Student’s t test | | |
| Types of mice: *Lrp2*^RPE^ | | **p-values: comparison between:** |
| Control | KO | Control and KO |
| Min/*P*_25_/Median/ *P*_75_/Max | Min/*P*_25_/Median/ *P*_75_/Max |  |
| 61.946/76.915/86.518/91.670/98.148 | 37.794/40.895/49.128/57.694/81.161 | **<0.0001** |
| Min: minimum. *P*_25_: 25^th^ percentile. *P*_75_: 75^th^ percentile. Max: Maximum. | | |

| Table S6: Numeric data for mRNA expression: Figure 2F | | | |
| --- | --- | --- | --- |
| 2-tails Student’s t test | | | |
| Gene | **Types of mice: *Lrp2*^RPE^** | | **p-values: comparison between:** |
|  | Control | KO | Control and KO |
|  | Min/*P*_25_/Median/ *P*_75_/Max | Min/*P*_25_/Median/ *P*_75_/Max |  |
| *Lrp2* | 0.849/0.859/1.045/1.118/1.172 | 0.339/0.343/0.446/0.500/0.503 | **<0.0001** |
| *Vegf* | 0.798/0.856/0.918/1.185/1.302 | 0.333/0.379/0.503/0.558/0.579 | **0.000646** |
| Min: minimum. *P*_25_: 25^th^ percentile. *P*_75_: 75^th^ percentile. Max: Maximum. | | | |

| Table S7: Numeric data for choroidal thickness: Figure 4C | | | |
| --- | --- | --- | --- |
| 2-tails Student’s t test | | | |
| Age(week) | **Types of mice**  ***Vegf*^RPE^** | | **p-values: comparison between:** |
|  | Control | KO | Control and KO |
|  | Min/*P*_25_/Median/ *P*_75_/Max | Min/*P*_25_/Median/ *P*_75_/Max |  |
| 3 | 9.560/10.093/10.895/11.363/11.900 | 8.520/9.895/11.185/11.358/11.470 | 0.812 |
| 4 | 10.010/10.890/11.310/12.100/13.090 | 6.710/8.410/10.395/11.525/12.390 | **0.045** |
| 5 | 10.060/10.945/11.840/12.470/13.120 | 6.970/9.130/10.745/11.750/13.110 | 0.107 |
| 6 | 10.900/11.240/11.580/12.965/13.500 | 7.100/8.395/10.870/11.730/11.900 | **0.013** |
| 7 | 10.630/11.365/11.950/13.905/14.090 | 7.850/8.870/10.065/10.770/11.160 | **<0.001** |
| 8 | 10.730/11.435/11.870/14.035/16.980 | 7.770/8.735/9.060/10.433/10.680 | **<0.001** |
| 9 | 10.370/11.195/12.010/14.585/15.620 | 7.980/9.148/10.105/10.615/11.540 | **0.001** |
| 10 | 11.760/12.140/12.930/15.160/15.510 | 8.170/9.225/10.310/11.238/11.470 | **<0.001** |
| Min: minimum. *P*_25_: 25^th^ percentile. *P*_75_: 75^th^ percentile. Max: Maximum. | | | |

| Table S8: Numeric data for axial length: Figure 4D | | | |
| --- | --- | --- | --- |
| 2-tails Student’s t test | | | |
| Age(week) | **Types of mice**  ***Vegf*^RPE^** | | **p-values: comparison between:** |
|  | Control | KO | Control and KO |
|  | Min/*P*_25_/Median/ *P*_75_/Max | Min/*P*_25_/Median/ *P*_75_/Max |  |
| 3 | 3.056/3.062/3.092/3.144/3.177 | 3.067/3.098/3.149/3.156/3.159 | 0.185 |
| 4 | 3.071/3.142/3.170/3.198/3.230 | 3.138/3.179/3.216/3.233/3.244 | **0.031** |
| 5 | 3.189/3.219/3.244/3.264/3.294 | 3.229/3.262/3.283/3.288/3.321 | **0.010** |
| 6 | 3.217/3.251/3.279/3.290/3.321 | 3.279/3.298/3.321/3.335/3.342 | **0.002** |
| 7 | 3.279/3.286/3.307/3.345/3.361 | 3.314/3.345/3.368/3.380/3.391 | **0.002** |
| 8 | 3.302/3.328/3.337/3.363/3.392 | 3.315/3.374/3.400/3.413/3.456 | **0.004** |
| 9 | 3.328/3.342/3.363/3.401/3.434 | 3.385/3.400/3.416/3.431/3.452 | **0.003** |
| 10 | 3.353/3.370/3.387/3.417/3.434 | 3.401/3.419/3.441/3.457/3.462 | **0.001** |
| Min: minimum. *P*_25_: 25^th^ percentile. *P*_75_: 75^th^ percentile. Max: Maximum. | | | |

| Table S9: Numeric data for vitreous chamber depth: Figure 4E | | | |
| --- | --- | --- | --- |
| 2-tails Student’s t test | | | |
| Age(week) | **Types of mice**  ***Vegf*^RPE^** | | **p-values: comparison between:** |
|  | Control | KO | Control and KO |
|  | Min/*P*_25_/Median/ *P*_75_/Max | Min/*P*_25_/Median/ *P*_75_/Max |  |
| 3 | 0.730/0.743/0.764/0.781/0.787 | 0.753/0.770/0.779/0.799/0.828 | 0.060 |
| 4 | 0.719/0.729/0.733/0.752/0.756 | 0.712/0.729/0.748/0.800/0.876 | 0.136 |
| 5 | 0.705/0.708/0.917/0.725/0.737 | 0.703/0.731/0.740/0.777/0.850 | **0.028** |
| 6 | 0.677/0.688/0.692/0.708/0.712 | 0.703/0.714/0.726/0.758/0.823 | **0.007** |
| 7 | 0.657/0.668/0.684/0.701/0.705 | 0.657/0.694/0.709/0.755/0.797 | **0.020** |
| 8 | 0.630/0.658/0.671/0.688/0.698 | 0.664/0.670/0.695/0.751/0.790 | **0.034** |
| 9 | 0.638/0.654/0.657/0.668/0.671 | 0.664/0.671/0.685/0.726/0.764 | **0.004** |
| 10 | 0.634/0.644/0.657/0.668/0.671 | 0.644/0.653/0.671/0.737/0.750 | **0.033** |
| Min: minimum. *P*_25_: 25^th^ percentile. *P*_75_: 75^th^ percentile. Max: Maximum. | | | |

| Table S10: Numeric data for refraction: Figure 4F | | | |
| --- | --- | --- | --- |
| 2-tails Student’s t test | | | |
| Age(week) | **Types of mice**  ***Vegf*^RPE^** | | **p-values: comparison between:** |
|  | Control | KO | Control and KO |
|  | Min/*P*_25_/Median/ *P*_75_/Max | Min/*P*_25_/Median/ *P*_75_/Max |  |
| 3 | -21.370/-18.780/0.460/1.218/3.320 | -23.720/-15.365/-1.725/1.940/4.990 | 0.912 |
| 4 | -2.270/-1.840/1.000/4.550/6.800 | -23.970/-19.135/-10.860/-3.685/3.320 | **0.001** |
| 5 | -7.020/-4.595/0.820/3.595/7.680 | -26.610/-19.485/-10.875/-1.490/5.450 | **0.014** |
| 6 | -0.540/2.460/5.190/10.555/12.330 | -20.990/-17.340/-5.720/0.465/2.660 | **0.001** |
| 7 | 3.410/5.450/6.860/11.850/13.920 | -24.220/-20.250/-11.560/3.200/7.690 | **0.003** |
| 8 | -4.620/-1.760/0.890/9.200/15.610 | -17.610/-16.090/-12.720/-1.000/-0.190 | **0.008** |
| 9 | 0.790/3.660/4.630/16.340/21.120 | -18.610/-13.630/-11.520/-1.223/2.910 | **0.002** |
| 10 | -2.680/-2.240/5.160/10.350/11.790 | -12.290/-11.885/-4.830/1.728/6.460 | **0.043** |
| Min: minimum. *P*_25_: 25^th^ percentile. *P*_75_: 75^th^ percentile. Max: Maximum. | | | |

| Table S11: Numeric data for choroidal thickness: Figure 4G | | | |
| --- | --- | --- | --- |
| 2-tails Student’s t test | | | |
| Age(week) | **Types of mice**  ***Vhl*^RPE^** | | **p-values: comparison between:** |
|  | Control | KO | Control and KO |
|  | Min/*P*_25_/Median/ *P*_75_/Max | Min/*P*_25_/Median/ *P*_75_/Max |  |
| 3 | 7.006/7.006/8.121/8.280/8.280 | 7.325/7.564/7.962/8.280/8.600 | 0.644 |
| 4 | 7.643/8.121/8.280/8.440/8.917 | 8.280/8.280/8.600/8.917/9.554 | **<0.001** |
| 5 | 7.643/8.200/8.599/8.599/8.912 | 8.280/8.280/9.076/9.554/10.191 | **0.004** |
| 6 | 7.644/8.440/8.758/8.999/9.236 | 8.600/8.600/8.917/8.997/9.873 | 0.658 |
| 7 | 7.643/8.280/8.440/8.560/9.236 | 7.643/8.917/9.236/9.872/10.191 | **0.006** |
| 8 | 8.560/8.560/8.917/8.917/8.917 | 8.917/9.475/9.554/9.873/10.191 | **<0.001** |
| 9 | 7.961/8.440/8.600/8.917/9.873 | 8.600/8.838/9.395/9.873/10.191 | **0.005** |
| 10 | 7.325/8.041/8.440/8.679/9.554 | 8.280/8.838/9.236/9.634/10.191 | **0.036** |
| Min: minimum. *P*_25_: 25^th^ percentile. *P*_75_: 75^th^ percentile. Max: Maximum. | | | |

| Table S12: Numeric data for axial length: Figure 4H | | | |
| --- | --- | --- | --- |
| 2-tails Student’s t test | | | |
| Age(week) | **Types of mice**  ***Vhlf*^RPE^** | | **p-values: comparison between:** |
|  | Control | KO | Control and KO |
|  | Min/*P*_25_/Median/ *P*_75_/Max | Min/*P*_25_/Median/ *P*_75_/Max |  |
| 3 | 3.091/3.097/3.103/3.110/3.150 | 3.082/3.082/3.091/3.101/3.108 | **0.033** |
| 4 | 3.150/3.189/3.222/3.250/3.269 | 3.108/3.131/3.142/3.165/3.209 | **0.0002** |
| 5 | 3.209/3.263/3.277/3.286/3.294 | 3.159/3.212/3.218/3.230/3.243 | **<0.001** |
| 6 | 3.285/3.292/3.303/3.315/3.345 | 3.249/3.260/3.277/3.298/3.337 | **0.003** |
| 7 | 3.320/3.335/3.350/3.381/3.396 | 3.243/3.292/3.303/3.337/3.354 | **0.001** |
| 8 | 3.338/3.370/3.405/3.439/3.456 | 3.320/3.329/3.337/3.355/3.372 | **0.0001** |
| 9 | 3.337/3.401/3.422/3.458/3.473 | 3.277/3.326/3.346/3.390/3.396 | **0.0002** |
| 10 | 3.413/3.420/3.435/3.458/3.498 | 3.269/3.309/3.350/3.390/3.421 | **0.0002** |
| Min: minimum. *P*_25_: 25^th^ percentile. *P*_75_: 75^th^ percentile. Max: Maximum. | | | |

| Table S13: Numeric data for vitreous chamber depth: Figure 4I | | | |
| --- | --- | --- | --- |
| 2-tails Student’s t test | | | |
| Age(week) | **Types of mice**  ***Vhl*^RPE^** | | **p-values: comparison between:** |
|  | Control | KO | Control and KO |
|  | Min/*P*_25_/Median/ *P*_75_/Max | Min/*P*_25_/Median/ *P*_75_/Max |  |
| 3 | 0.739/0.746/0.756/0.773/0.790 | 0.705/0.733/0.750/0.757/0.773 | 0.099 |
| 4 | 0.714/0.735/0.748/0.756/0.765 | 0.673/0.680/0.705/0.733/0.747 | **0.001** |
| 5 | 0.688/0.697/0.710/0.722/0.756 | 0.663/0.680/0.684/0.716/0.722 | 0.066 |
| 6 | 0.671/0.677/0.688/0.697/0.722 | 0.646/0.659/0.671/0.693/0.714 | 0.153 |
| 7 | 0.612/0.646/0.671/0.682/0.714 | 0.620/0.637/0.654/0.684/0.688 | 0.459 |
| 8 | 0.629/0.644/0.654/0.673/0.688 | 0.603/0.620/0.646/0.659/0.671 | 0.135 |
| 9 | 0.629/0.644/0.654/0.680/0.688 | 0.586/0.610/0.629/0.650/0.663 | **0.010** |
| 10 | 0.595/0.610/0.633/0.646/0.697 | 0.578/0.586/0.620/0.637/0.646 | 0.139 |
| Min: minimum. *P*_25_: 25^th^ percentile. *P*_75_: 75^th^ percentile. Max: Maximum. | | | |

| Table S14: Numeric data for refraction: Figure 4J | | | |
| --- | --- | --- | --- |
| 2-tails Student’s t test | | | |
| Age(week) | **Types of mice**  ***Vhl*^RPE^** | | **p-values: comparison between:** |
|  | Control | KO | Control and KO |
|  | Min/*P*_25_/Median/ *P*_75_/Max | Min/*P*_25_/Median/ *P*_75_/Max |  |
| 3 | -6.710/-5.975/-3.120/3.263/6.930 | -15.570/-11.573/-7.755/0.655/8.550 | 0.169 |
| 4 | -8.570/-4.305/0.005/4.653/8.670 | -3.480/0.800/2.785/3.868/4.630 | 0.260 |
| 5 | -7.360/0.988/4.015/6.763/16.320 | -0.780/2.485/3.350/6.080/6.940 | 0.871 |
| 6 | -4.410/-0.758/2.805/8.355/11.270 | -2.570/-0.608/0.805/4.755/7.080 | 0.306 |
| 7 | 0.260/1.420/4.155/10.440/11.410 | -0.980/3.380/7.890/9.700/10.440 | 0.569 |
| 8 | -1.530/1.300/5.015/7.563/10.410 | 0.500/2.983/4.200/6.175/7.210 | 0.869 |
| 9 | 1.060/2.468/4.810/8.033/11.470 | 1.480/2.745/4.585/9.200/11.070 | 0.907 |
| 10 | -4.830/0.300/3.705/7.423/10.990 | -2.360/1.143/3.645/5.808/9.930 | 0.967 |
| Min: minimum. *P*_25_: 25^th^ percentile. *P*_75_: 75^th^ percentile. Max: Maximum. | | | |

| Table S15: Numeric data for baseline characteristics of high myopia patients | | | |
| --- | --- | --- | --- |
| 2-tails Student’s t test | | | |
|  | **Choriocapillaris** | | **p-values: comparison between:** |
|  | + | - |  |
| Number | 5 (1male, 4 female) | 8 (4 male, 4 female) |  |
|  | Min/*P*_25_/Median/ *P*_75_/Max | Min/*P*_25_/Median/ *P*_75_/Max | + and - |
| Age | 65.000/65.000/69.000/70.000/70.000 | 67.000/69.250/70.000/70.750/74.000 | 0.092 |
| BCVA | 1.000/1.000/1.200/1.200/1.200 | 0.900/0.900/1.200/1.200/1.200 | 0.692 |
| logMAR | -0.080/-0.080/-0.080/0.000/0.000 | -0.080/-0.080/-0.080/0.050/0.050 | 0.633 |
| SE | -9.000/-8.250/-7.250/-0.250/-0.250 | -16.250/-14.625/-9.250/-6.625/-0.875 | 0.093 |
| IOP | 13.000/13.000/13.000/14.850/15.000 | 11.000/11.475/12.000/13.000/14.000 | **0.027** |
| AL | 27.140/27.180/27.310/27.850/28.290 | 26.660/27.738/28.800/29.638/30.370 | 0.054 |
| Choroidal thickness | 67.500/78.000/122.500/158.250/162.500 | 14.500/29.000/40.750/68.000/74.500 | **0.001** |
| BCVA: Best Corrected Visual Acuity. logMAR: acronym for the Logarithm of the Minimum Angle of Resolution. SE: spherical equivalent. IOP: intraocular pressure. Min: minimum. *P*_25_: 25^th^ percentile. *P*_75_: 75^th^ percentile. Max: Maximum. | | | |

| Table S16: Numeric data for variation in 6-month follow-up: Figure 5B | | | |
| --- | --- | --- | --- |
| 2-tails Student’s t test | | | |
|  | **Choriocapillaris** | | **p-values: comparison between:** |
|  | + | - |  |
|  | Min/*P*_25_/Median/ *P*_75_/Max | Min/*P*_25_/Median/ *P*_75_/Max | + and - |
| ΔBCVA | -0.500/-0.350/0.000/0.200/0.200 | 0.000/0.000/0.000/0.000/0.100 | 0.497 |
| ΔlogMAR | -0.080/-0.080/0.000/0.155/0.230 | -0.050/0.000/0.000/0.000/0.000 | 0.442 |
| ΔSE | 0.000/0.000/0.000/0.000/0.000 | -2.500/-0.781/-0.125/0.000/0.000 | 0.215 |
| ΔIOP | -2.700/-1.350/1.000/2.000/2.000 | -1.000/-0.450/0.500/1.000/3.000 | 0.943 |
| ΔAL | 0.000/0.000/0.000/0.005/0.010 | 0.010/0.020/0.035/0.060/0.130 | **0.029** |
| ΔChoroidal thickness | -16.500/-9.000/2.500/24.500/25.500 | -19.000/-4.250/1.250/7.000/10.000 | 0.353 |
| BCVA: Best Corrected Visual Acuity. logMAR: acronym for the Logarithm of the Minimum Angle of Resolution. SE: spherical equivalent. IOP: intraocular pressure. Min: minimum. *P*_25_: 25^th^ percentile. *P*_75_: 75^th^ percentile. Max: Maximum. | | | |

| Table S17: Numeric data for axial length: Figure S2B | | | | |
| --- | --- | --- | --- | --- |
| One-way repeated measures ANOVA: F(2, 15) = 2.033, p=0.1654 | | | | |
| Types of mice | Min/*P*_25_/Median/ *P*_75_/Max | **p-values: comparison between:** | | |
|  |  | different types | | |
| *Lrp2*^floxed/-^ *Chx10*+ | 3.337/3.360/3.381/3.387/3.392 | with |  |  |
| *Lrp2*^floxed/floxed^*Chx10*- | 3.337/3.376/3.399/3.444/3.457 | 0.421 | with |  |
| *Lrp2*^floxed/floxed^ *Chx10*+ | 3.264/3.299/3.322/3.414/3.419 | 0.340 | 0.066 | with |
| Min: minimum. *P*_25_: 25^th^ percentile. *P*_75_: 75^th^ percentile. Max: Maximum. | | | | |

| Table S18: Numeric data for axial length: Figure S4B | | | | |
| --- | --- | --- | --- | --- |
| One-way repeated measures ANOVA: F(2, 17) = 5.106, p=0.0183 | | | | |
| Types of mice | Min/*P*_25_/Median/ *P*_75_/Max | **p-values: comparison between:** | | |
|  |  | different types | | |
| *Lrp2*^floxed/-^ Best1+ | 3.296/3.299/3.314/3.349/3.359 | with |  |  |
| *Lrp2*^floxed/floxed^ Best1- | 3.310/3.313/3.343/3.365/3.392 | 0.972 | with |  |
| *Lrp2*^floxed/floxed^ Best1+ | 4.237/4.455/4.648/4.758/5.042 | **<0.0001** | **<0.0001** | with |
| Min: minimum. *P*_25_: 25^th^ percentile. *P*_75_: 75^th^ percentile. Max: Maximum. | | | | |

| Table S19: Numeric data for ocular parameters difference: Figure S4C | | | |
| --- | --- | --- | --- |
| 2-tails Student’s t test | | | |
| Ocular parameters | **Types of mice:** | | **p-values: comparison between:** |
|  | *Lrp2*^floxed/-^ Best1+ | *Lrp2*^floxed/floxed^ Best1- | Control and KO |
|  | Min/*P*_25_/Median/ *P*_75_/Max | Min/*P*_25_/Median/ *P*_75_/Max |  |
| Corneal thickness | 0.092/0.094/0.101/0.103/0.103 | 0.091/0.099/0.100/0.103/0.105 | 0.790 |
| AC depth | 0.322/0.331/0.362/0.374/0.377 | 0.321/0.324/0.350/0.359/0.384 | 0.545 |
| Lens thickness | 1.922/1.925/1.946/1.960/1.961 | 1.947/1.965/2.003/2.039/2.066 | 0.057 |
| VC depth + retinal thickness | 0.853/0.859/0.879/0.879/0.879 | 0.802/0.812/0.850/0.884/0.890 | 0.271 |
| AC: anterior chamber. VC: vitreous chamber. Min: minimum. *P*_25_: 25^th^ percentile. *P*_75_: 75^th^ percentile. Max: Maximum. | | | |

| Table S20: Numeric data for retinal thickness: Figure S5A | | | |
| --- | --- | --- | --- |
| 2-tails Student’s t test | | | |
| Age(week) | **Types of mice**  ***Lrp2*^RPE^** | | **p-values: comparison between:** |
|  | Control | KO | Control and KO |
|  | Min/*P*_25_/Median/ *P*_75_/Max | Min/*P*_25_/Median/ *P*_75_/Max |  |
| 3 | 92.120/108.885/127.435/149.108/188.340 | 43.090/57.058/64.940/75.523/100.280 | **<0.0001** |
| 6 | 77.090/130.013/155.505/167.710/197.060 | 41.810/54.365/65.640/79.668/118.500 | **<0.0001** |
| 8 | 98.200/136.210/148.535/164.893/187.610 | 30.000/47.353/58.480/70.825/106.560 | **<0.0001** |
| Min: minimum. *P*_25_: 25^th^ percentile. *P*_75_: 75^th^ percentile. Max: Maximum. | | | |

| Table S21: Numeric data for retinal ONL thickness: Figure S5B | | | |
| --- | --- | --- | --- |
| 2-tails Student’s t test | | | |
| Age(week) | **Types of mice**  ***Lrp2*^RPE^** | | **p-values: comparison between:** |
|  | Control | KO | Control and KO |
|  | Min/*P*_25_/Median/ *P*_75_/Max | Min/*P*_25_/Median/ *P*_75_/Max |  |
| 3 | 26.060/32.250/38.520/47.270/58.900 | 10.400/17.480/20.000/24.335/33.610 | **<0.0001** |
| 6 | 20.400/30.075/37.000/42.853/52.300 | 7.080/14.193/17.515/20.953/32.130 | **<0.0001** |
| 8 | 25.730/32.168/36.050/39.598/49.320 | 6.580/10.610/13.185/15.978/21.090 | **<0.0001** |
| Min: minimum. *P*_25_: 25^th^ percentile. *P*_75_: 75^th^ percentile. Max: Maximum. | | | |

| Table S22: Numeric data for retinal INL thickness: Figure S5C | | | |
| --- | --- | --- | --- |
| 2-tails Student’s t test | | | |
| Age(week) | **Types of mice**  ***Lrp2*^RPE^** | | **p-values: comparison between:** |
|  | Control | KO | Control and KO |
|  | Min/*P*_25_/Median/ *P*_75_/Max | Min/*P*_25_/Median/ *P*_75_/Max |  |
| 3 | 14.710/20.558/24.795/30.302/42.190 | 6.240/8.240/10.060/12.728/21.470 | **<0.0001** |
| 6 | 9.160/21.620/28.295/34.280/43.760 | 4.160/9.710/11.805/14.790/27.040 | **<0.0001** |
| 8 | 12.510/22.390/25.225/29.613/34.300 | 4.160/8.278/11.280/13.150/20.900 | **<0.0001** |
| Min: minimum. *P*_25_: 25^th^ percentile. *P*_75_: 75^th^ percentile. Max: Maximum. | | | |

| Table S23: Numeric data for scleral thickness: Figure S5D | | | |
| --- | --- | --- | --- |
| 2-tails Student’s t test | | | |
| Age(week) | **Types of mice**  ***Lrp2*^RPE^** | | **p-values: comparison between:** |
|  | Control | KO | Control and KO |
|  | Min/*P*_25_/Median/ *P*_75_/Max | Min/*P*_25_/Median/ *P*_75_/Max |  |
| 3 | 4.740/9.655/11.430/15.810/21.210 | 4.160/6.510/7.810/9.613/13.410 | **<0.0001** |
| 6 | 5.420/9.678/12.180/15.190/27.900 | 6.580/10.368/12.445/15.030/22.630 | 0.955 |
| 8 | 6.580/11.913/15.480/19.225/31.050 | 5.010/9.490/10.850/12.488/18.350 | **<0.0001** |
| Min: minimum. *P*_25_: 25^th^ percentile. *P*_75_: 75^th^ percentile. Max: Maximum. | | | |

| Table S24: Numeric data for retinal vessels: Figure S6B | | | |
| --- | --- | --- | --- |
| 2-tails Student’s t test | | | |
|  | **Types of mice: *Lrp2*^RPE^** | | **p-values: comparison between:** |
|  | Control | KO | Control and KO |
|  | Mean/SD | Mean/SD |  |
| No.Dendrite Segments/area(mm^2^) | 1779.595/223.185 | 382.915/225.767 | **0.002** |
| No.Dendrite Branches/area(mm^2^) | 6.819/1.564 | 2.548/1.263 | **0.021** |
| SD: standard deviation. | | | |

| Table S25: Numeric data for scotopic ERG a-wave amplitude: Figure S7B | | | |
| --- | --- | --- | --- |
| 2-tails Student’s t test | | | |
| Stimulus  Intensity  (cd/m^2^) | **Types of mice**  ***Lrp2*^RPE^** | | **p-values: comparison between:** |
|  | Control | KO | Control and KO |
|  | Min/*P*_25_/Median/ *P*_75_/Max | Min/*P*_25_/Median/ *P*_75_/Max |  |
| 0.00005 | 0.000/0.000/1.550/5.050/5.700 | 0.000/0.240/1.430/7.473/9.330 | 0.750 |
| 0.0004 | 0.000/0.000/2.230/5.870/6.340 | 1.030/1.505/3.805/4.973/5.070 | 0.708 |
| 0.001 | 1.600/1.813/3.260/4.318/4.400 | 0.000/0.213/1.205/2.895/3.340 | 0.132 |
| 0.005 | 0.000/0.245/4.110/23.980/29.560 | 0.000/0.445/7.250/21.548/24.490 | 0.974 |
| 0.02 | 1.760/2.540/9.265/17.355/18.590 | 0.000/1.720/10.885/18.333/19.480 | 0.922 |
| 0.1 | 29.930/33.405/46.760/87.715/100.390 | 0.000/0.000/0.440/5.860/7.520 | **0.013** |
| 0.5 | 75.010/93.678/160.140/197.968/207.090 | 0.000/1.830/12.130/61.918/76.910 | **0.009** |
| 2 | 198.490/205.388/231.610/253.738/259.270 | 0.000/5.600/49.165/105.998/116.020 | **0.0009** |
| 10 | 287.480/289.290/296.710/335.735/348.080 | 4.410/17.688/70.980/92.120/94.680 | **<0.0001** |
| 50 | 304.650/312.905/360.825/433.218/449.630 | 38.200/53.993/107.430/142.830/152.610 | **0.0005** |
| Min: minimum. *P*_25_: 25^th^ percentile. *P*_75_: 75^th^ percentile. Max: Maximum. | | | |

| Table S26: Numeric data for scotopic ERG b-wave amplitude: Figure S7C | | | |
| --- | --- | --- | --- |
| 2-tails Student’s t test | | | |
| Stimulus  Intensity  (cd/m^2^) | **Types of mice**  ***Lrp2*^RPE^** | | **p-values: comparison between:** |
|  | Control | KO | Control and KO |
|  | Min/*P*_25_/Median/ *P*_75_/Max | Min/*P*_25_/Median/ *P*_75_/Max |  |
| 0.00005 | 22.270/22.665/24.935/45.903/52.530 | 1.190/1.758/5.650/12.565/14.140 | **0.019** |
| 0.0004 | 38.320/42.295/67.155/86.863/89.120 | 3.780/4.288/6.990/16.323/19.040 | **0.004** |
| 0.001 | 97.160/101.308/133.350/172.840/179.470 | 3.460/7.788/21.600/29.098/31.320 | **0.001** |
| 0.005 | 133.460/140.915/207.425/308.278/327.180 | 15.250/19.020/37.600/52.820/55.470 | **0.007** |
| 0.02 | 150.570/163.620/228.295/296.720/311.020 | 13.680/14.485/46.225/91.398/96.680 | **0.004** |
| 0.1 | 178.290/184.865/244.755/345.430/365.600 | 35.460/47.118/82.680/84.553/84.980 | **0.005** |
| 0.5 | 288.630/256.180/317.020/444.288/483.980 | 37.970/39.643/68.840/122.683/132.570 | **0.003** |
| 2 | 356.490/367.365/462.095/558.910/570.480 | 20.490/42.878/117.660/133.305/135.980 | **0.0007** |
| 10 | 378.780/412.278/534.560/654.810/687.630 | 63.420/65.420/95.075/125.818/128.180 | **0.0006** |
| 50 | 424.790/467.303/605.940/711.945/743.580 | 32.800/36.133/49.385/91.573/104.550 | **0.0002** |
| Min: minimum. *P*_25_: 25^th^ percentile. *P*_75_: 75^th^ percentile. Max: Maximum. | | | |

| Table S27: Numeric data for scotopic ERG a-wave latency: Figure S7D | | | |
| --- | --- | --- | --- |
| 2-tails Student’s t test | | | |
| Stimulus  Intensity  (cd/m^2^) | **Types of mice**  ***Lrp2*^RPE^** | | **p-values: comparison between:** |
|  | Control | KO | Control and KO |
|  | Min/*P*_25_/Median/ *P*_75_/Max | Min/*P*_25_/Median/ *P*_75_/Max |  |
| 0.00005 | 0.000/7.175/31.100/43.700/47.100 | 31.100/32.500/37.100/48.900/52.700 | 0.308 |
| 0.0004 | 25.500/25.700/35.100/43.900/43.900 | 23.100/25.500/39.900/67.425/74.200 | 0.474 |
| 0.001 | 20.800/23.175/31.100/34.900/35.900 | 29.500/29.500/39.100/52.300/53.500 | 0.186 |
| 0.005 | 27.900/28.500/32.300/39.100/40.700 | 31.900/34.900/44.300/54.900/58.300 | 0.110 |
| 0.02 | 29.500/29.700/31.500/36.900/38.300 | 30.300/33.500/43.150/44.925/45.500 | 0.097 |
| 0.1 | 27.100/27.300/29.100/34.500/35.900 | 31.100/33.700/44.300/55.500/58.300 | 0.056 |
| 0.5 | 24.700/24.900/26.700/31.500/32.700 | 35.900/36.100/37.100/40.500/41.500 | **0.003** |
| 2 | 23.100/23.300/24.700/29.100/30.300 | 32.700/32.900/33.900/34.900/35.100 | **0.002** |
| 10 | 21.500/21.500/22.300/26.100/27.100 | 29.500/29.700/30.700/33.500/34.300 | **0.003** |
| 50 | 20.000/20.375/21.500/25.100/26.300 | 28.700/29.500/33.500/35.100/35.100 | **0.002** |
| Min: minimum. *P*_25_: 25^th^ percentile. *P*_75_: 75^th^ percentile. Max: Maximum. | | | |

| Table S28: Numeric data for scotopic ERG b-wave latency: Figure S7E | | | |
| --- | --- | --- | --- |
| 2-tails Student’s t test | | | |
| Stimulus  Intensity  (cd/m^2^) | **Types of mice**  ***Lrp2*^RPE^** | | **p-values: comparison between:** |
|  | Control | KO | Control and KO |
|  | Min/*P*_25_/Median/ *P*_75_/Max | Min/*P*_25_/Median/ *P*_75_/Max |  |
| 0.00005 | 99.000/102.175/119.700/129.500/130.100 | 90.200/90.600/93.800/131.125/142.900 | 0.444 |
| 0.0004 | 114.900/115.300/120.500/128.700/130.100 | 86.200/92.175/114.500/124.300/126.100 | 0.279 |
| 0.001 | 97.400/97.800/99.400/115.325/120.500 | 89.400/92.800/109.350/118.100/118.900 | 0.776 |
| 0.005 | 87.800/88.200/89.400/93.000/94.200 | 94.200/94.800/97.400/142.450/157.200 | 0.212 |
| 0.02 | 59.900/60.300/65.050/80.000/83.800 | 95.000/97.000/104.550/112.100/114.100 | **0.001** |
| 0.1 | 66.200/67.200/75.400/89.000/91.800 | 95.000/98.975/117.300/133.900/137.300 | **0.010** |
| 0.5 | 52.700/52.900/56.300/73.425/78.200 | 78.200/85.575/123.300/155.400/160.900 | **0.019** |
| 2 | 53.500/56.275/65.800/76.600/79.800 | 81.400/85.200/111.350/146.425/153.200 | **0.029** |
| 10 | 51.900/55.275/68.600/91.000/97.400 | 96.900/97.625/101.800/124.725/131.700 | **0.026** |
| 50 | 68.600/69.000/78.200/104.125/110.100 | 59.900/69.275/98.600/103.325/104.500 | 0.655 |
| Min: minimum. *P*_25_: 25^th^ percentile. *P*_75_: 75^th^ percentile. Max: Maximum. | | | |

| Table S29: Numeric data for photopic ERG b-wave amplitude: Figure S7F | | | |
| --- | --- | --- | --- |
| 2-tails Student’s t test | | | |
| Stimulus  Intensity  (cd/m^2^) | **Types of mice**  ***Lrp2*^RPE^** | | **p-values: comparison between:** |
|  | Control | KO | Control and KO |
|  | Min/*P*_25_/Median/ *P*_75_/Max | Min/*P*_25_/Median/ *P*_75_/Max |  |
| 4 | 74.380/74.928/82.315/105.520/111.340 | 3.060/4.668/11.725/20.575/22.780 | **0.0002** |
| 10 | 125.410/125.478/128.525/141.420/144.770 | 0.000/2.823/12.265/15.370/16.080 | **<0.0001** |
| 20 | 147.340/147.423/155.660/183.900/190.650 | 0.000/3.810/16.120/21.050/22.400 | **<0.0001** |
| Min: minimum. *P*_25_: 25^th^ percentile. *P*_75_: 75^th^ percentile. Max: Maximum. | | | |

| Table S30: Numeric data for photopic ERG b-wave latency: Figure S7G | | | |
| --- | --- | --- | --- |
| 2-tails Student’s t test | | | |
| Stimulus  Intensity  (cd/m^2^) | **Types of mice**  ***Lrp2*^RPE^** | | **p-values: comparison between:** |
|  | Control | KO | Control and KO |
|  | Min/*P*_25_/Median/ *P*_75_/Max | Min/*P*_25_/Median/ *P*_75_/Max |  |
| 4 | 44.700/44.700/45.900/51.900/53.500 | 56.700/62.075/84.200/116.525/125.300 | **0.033** |
| 10 | 45.500/45.900/48.700/59.825/63.000 | 76.600/76.800/80.600/106.525/114.100 | **0.009** |
| 20 | 47.100/47.500/50.300/61.425/64.600 | 61.500/62.875/71.400/80.000/81.400 | **0.021** |
| Min: minimum. *P*_25_: 25^th^ percentile. *P*_75_: 75^th^ percentile. Max: Maximum. | | | |

| Table S31: Numeric data for refraction: Figure S8A | | | | |
| --- | --- | --- | --- | --- |
| One-way repeated measures ANOVA: F(2, 12) = 18.64, p=0.0002 | | | | |
| Types of mice | Min/*P*_25_/Median/ *P*_75_/Max | **p-values: comparison between:** | | |
|  | 3 weeks | different types | | |
| LIM | -1.330/-1.010/-0.200/0.420/0.910 | with |  |  |
| *Vegf* KO vs.*Vegf* Cont | -23.720/-15.365/-1.725/1.940/4.990 | 0.403 | with |  |
| *Vhl* KO vs. *Vhl* Cont | -15.570/-11.573/-4.965/7.455/11.140 | 0.724 | 0.854 | with |
|  | 4 weeks | different types | | |
| LIM | -11.530/-11.275/-8.530/-6.785/-5.100 | with |  |  |
| *Vegf* KO vs.*Vegf* Cont | -23.970/-19.135/-10.860/-3.685/3.320 | 0.797 | with |  |
| *Vhl* KO vs. *Vhl* Cont | -3.480/-0.030/2.080/3.375/4.630 | **0.0002** | **0.005** | with |
|  | 5 weeks | different types | | |
| LIM | -15.710/-15.380/-6.330/-2.250/0.000 | with |  |  |
| *Vegf* KO vs.*Vegf* Cont | -26.610/-19.485/-10.875/-1.490/5.450 | 0.874 | with |  |
| *Vhl* KO vs. *Vhl* Cont | 2.570/3.313/4.250/6.610/7.620 | **0.026** | **0.004** | with |
|  | 6 weeks | different types | | |
| LIM | -20.630/-16.485/-5.050/-1.395/2.250 | with |  |  |
| *Vegf* KO vs.*Vegf* Cont | -20.990/-17.340/-5.720/0.465/2.660 | 0.999 | with |  |
| *Vhl* KO vs. *Vhl* Cont | -1.800/-0.158/3.225/5.460/9.780 | 0.086 | **0.009** | with |
|  | 7 weeks | different types | | |
| LIM | -22.300/-18.255/-12.970/-10.800/-9.430 | with |  |  |
| *Vegf* KO vs.*Vegf* Cont | -24.220/-20.250/-11.560/-3.200/7.690 | 0.528 | with |  |
| *Vhl* KO vs. *Vhl* Cont | -0.980/1.513/7.350/8.575/10.440 | **0.0003** | **0.040** | with |
|  | 8 weeks | different types | | |
| LIM | -15.130/-15.070/-6.080/-5.610/-5.510 | with |  |  |
| *Vegf* KO vs.*Vegf* Cont | -17.610/-16.090/-12.720/-1.000/-0.190 | 0.996 | with |  |
| *Vhl* KO vs. *Vhl* Cont | 0.500/2.428/3.105/4.685/5.280 | **0.009** | **0.011** | with |
|  | 9 weeks | different types | | |
| LIM | -13.790/-13.360/-12.720/-7.800/-6.380 | with |  |  |
| *Vegf* KO vs.*Vegf* Cont | -18.610/-13.630/-11.520/-1.223/2.910 | 0.816 | with |  |
| *Vhl* KO vs. *Vhl* Cont | 1.330/1.878/3.785/5.935/11.070 | **<0.0001** | **0.016** | with |
|  | 10 weeks | different types | | |
| LIM | -12.100/-11.250/-9.850/-8.435/-8.360 | with |  |  |
| *Vegf* KO vs.*Vegf* Cont | -12.290/-11.885/-4.830/1.728/6.460 | 0.260 | with |  |
| *Vhl* KO vs. *Vhl* Cont | -2.360/1.143/3.645/5.808/9.930 | **<0.0001** | 0.087 | with |
| Min: minimum. *P*_25_: 25^th^ percentile. *P*_75_: 75^th^ percentile. Max: Maximum. | | | | |

| Table S32: Numeric data for change of choroidal thickness: Figure S8B | | | | |
| --- | --- | --- | --- | --- |
| One-way repeated measures ANOVA: F(2, 12) = 13.86, p=0.0008 | | | | |
| Types of mice | Min/*P*_25_/Median/ *P*_75_/Max | **p-values: comparison between:** | | |
|  | 3 weeks | different types | | |
| LIM | -0.980/-0.635.-0.200/0.370/0.470 | with |  |  |
| *Vegf* KO vs.*Vegf* Cont | -0.600/-0.590/-0.010/0.875/1.300 | 0.829 | with |  |
| *Vhl* KO vs. *Vhl* Cont | -0.637/-0.637/0.000/0.796/1.274 | 0.881 | 0.995 | with |
|  | 4 weeks | different types | | |
| LIM | -1.310/-1.165/-0.840/-0.305/-0.140 | with |  |  |
| *Vegf* KO vs.*Vegf* Cont | -4.050/-2.910/-0.700/0.400/0.650 | 0.896 | with |  |
| *Vhl* KO vs. *Vhl* Cont | -0.318/-0.200/0.637/1.115/1.274 | **0.013** | 0.221 | with |
|  | 5 weeks | different types | | |
| LIM | -2.010/-1.960/-1.740/-0.700/-0.450 | with |  |  |
| *Vegf* KO vs.*Vegf* Cont | -5.050/-4.160/-0.350/0.620/0.780 | 0.998 | with |  |
| *Vhl* KO vs. *Vhl* Cont | -0.318/-0.159/0.318/1.115/1.592 | 0.008 | 0.324 | with |
|  | 6 weeks | different types | | |
| LIM | -2.650/-2.415/-1.760/-0.370/0.400 | with |  |  |
| *Vegf* KO vs.*Vegf* Cont | -4.300/-3.710/-1.820/-0.940/-0.550 | 0.661 | with |  |
| *Vhl* KO vs. *Vhl* Cont | -0.637/-0.159/0.318/0.786/0.955 | 0.054 | **0.034** | with |
|  | 7 weeks | different types | | |
| LIM | -2.630/-2.485/-1.290/-0.270/-0.140 | with |  |  |
| *Vegf* KO vs.*Vegf* Cont | -4.370/-4.255/-2.930/-1.700/-0.700 | 0.190 | with |  |
| *Vhl* KO vs. *Vhl* Cont | -0.955/-0.637/0.318/1.274/1.592 | 0.086 | **0.010** | with |
|  | 8 weeks | different types | | |
| LIM | -3.080/-2.775/-1.190/-0.525/0.000 | with |  |  |
| *Vegf* KO vs.*Vegf* Cont | -8.090/-6.175/-3.890/-1.735/-0.970 | 0.245 | with |  |
| *Vhl* KO vs. *Vhl* Cont | 0.000/0.159/0.637/0.637/0.637 | **0.043** | **0.044** | with |
|  | 9 weeks | different types | | |
| LIM | -3.060/-2.705/-2.180/-0.515/-0.340 | with |  |  |
| *Vegf* KO vs.*Vegf* Cont | -5.210/-4.565/-3.190/-1.160/-1.090 | 0.453 | with |  |
| *Vhl* KO vs. *Vhl* Cont | 0.000/0.318/0.955/1.115/1.274 | **0.013** | **0.017** | with |
|  | 10 weeks | different types | | |
| LIM | -3.520/-3.385/-2.480/-0.195/0.380 | with |  |  |
| *Vegf* KO vs.*Vegf* Cont | -5.540/-4.925/-3.810/-1.055/-0.280 | 0.585 | with |  |
| *Vhl* KO vs. *Vhl* Cont | 0.637/0.637/0.955/1.115/1.274 | **0.041** | **0.026** | with |
| Min: minimum. *P*_25_: 25^th^ percentile. *P*_75_: 75^th^ percentile. Max: Maximum. | | | | |

| Table S33: Numeric data for change of axial length: Figure S8C | | | | |
| --- | --- | --- | --- | --- |
| One-way repeated measures ANOVA: F(2, 22) = 12.29, p=0.0003 | | | | |
| Types of mice | Min/*P*_25_/Median/ *P*_75_/Max | **p-values: comparison between:** | | |
|  | 3 weeks | different types | | |
| LIM | -0.028/-0.018/0.000/0.022/0.036 | with |  |  |
| *Vegf* KO vs.*Vegf* Cont | -0.032/-0.014/0.026/0.039/0.046 | 0.459 | with |  |
| *Vhl* KO vs. *Vhl* Cont | -0.093/-0.081/-0.050/0.004/0.008 | 0.100 | **0.049** | with |
|  | 4 weeks | different types | | |
| LIM | -0.014/0.004/0.028/0.043/0.057 | with |  |  |
| *Vegf* KO vs.*Vegf* Cont | 0.016/0.022/0.032/0.107/0.137 | 0.223 | with |  |
| *Vhl* KO vs. *Vhl* Cont | -0.161/-0.144/-0.110/-0.034/0.025 | **0.017** | **0.005** | with |
|  | 5 weeks | different types | | |
| LIM | 0.000/0.008/0.021/0.033/0.043 | with |  |  |
| *Vegf* KO vs.*Vegf* Cont | 0.005/0.011/0.042/0.095/0.099 | 0.196 | with |  |
| *Vhl* KO vs. *Vhl* Cont | -0.160/-0.148/-0.076/-0.012/-0.006 | **0.030** | **0.001** | with |
|  | 6 weeks | different types | | |
| LIM | 0.007/0.015/0.042/0.109/0.155 | with |  |  |
| *Vegf* KO vs.*Vegf* Cont | 0.012/0.021/0.042/0.098/0.118 | 0.947 | with |  |
| *Vhl* KO vs. *Vhl* Cont | -0.144/-0.128/-0.054/-0.039/-0.026 | **0.004** | **0.002** | with |
|  | 7 weeks | different types | | |
| LIM | 0.028/0.030/0.035/0.052/0.065 | with |  |  |
| *Vegf* KO vs.*Vegf* Cont | 0.023/0.034/0.056/0.077/0.077 | 0.236 | with |  |
| *Vhl* KO vs. *Vhl* Cont | -0.102/-0.098/-0.077/-0.013/0.025 | **0.010** | **0.005** | with |
|  | 8 weeks | different types | | |
| LIM | 0.000/0.011/0.021/0.056/0.061 | with |  |  |
| *Vegf* KO vs.*Vegf* Cont | -0.006/0.004/0.029/0.099/0.121 | 0.549 | with |  |
| *Vhl* KO vs. *Vhl* Cont | -0.179/-0.139/-0.040/-0.026/-0.025 | **0.021** | **0.013** | with |
|  | 9 weeks | different types | | |
| LIM | 0.014/0.018/0.035/0.043/0.050 | with |  |  |
| *Vegf* KO vs.*Vegf* Cont | -0.007/0.009/0.077/0.084/0.089 | 0.333 | with |  |
| *Vhl* KO vs. *Vhl* Cont | -0.153/-0.145/-0.128/-0.017/0.000 | **0.015** | **0.006** | with |
|  | 10 weeks | different types | | |
| LIM | 0.000/0.001/0.036/0.046/0.049 | with |  |  |
| *Vegf* KO vs.*Vegf* Cont | 0.028/0.032/0.058/0.070/0.075 | 0.095 | with |  |
| *Vhl* KO vs. *Vhl* Cont | -0.178/-0.136/-0.035/-0.011/-0.004 | **0.043** | **0.019** | with |
| Min: minimum. *P*_25_: 25^th^ percentile. *P*_75_: 75^th^ percentile. Max: Maximum. | | | | |
